# The aberrant tonsillar microbiota modulates autoimmune responses in rheumatoid arthritis

**DOI:** 10.1172/jci.insight.175916

**Published:** 2024-08-20

**Authors:** Jing Li, Shenghui Li, Jiayang Jin, Ruochun Guo, Yuebo Jin, Lulu Cao, Xuanlin Cai, Peishi Rao, Yan Zhong, Xiaohong Xiang, Xiaolin Sun, Jianping Guo, Fanlei Hu, Hua Ye, Yuan Jia, Wenjing Xiao, Yuan An, Xuan Zhang, BinBin Xia, Rentao Yang, Yuanjie Zhou, Lijun Wu, Junjie Qin, Jing He, Jun Wang, Zhanguo Li

**Affiliations:** 1 Department of Rheumatology and Immunology, Peking University People’s Hospital, Beijing, China.; 2 Beijing Key Laboratory for Rheumatism Mechanism and Immune Diagnosis (BZ0135), Beijing, China.; 3 Key Laboratory of Precision Nutrition and Food Quality, Department of Nutrition and Health, China Agricultural University, Beijing, China.; 4 Puensum Genetech Institute, Wuhan, China.; 5 Department of Rheumatology and Immunology, The People’s Hospital of Xin Jiang Uygur Autonomous Region, Urumqi, China.; 6 Emergency Department, Peking University People’s Hospital, Beijing, China.; 7 CAS Key Laboratory for Pathogenic Microbiology and Immunology, Institute of Microbiology, Chinese Academy of Sciences, Beijing, China.; 8 University of Chinese Academy of Sciences, Beijing, China.; 9 Promegene Translational Research Institute, Shenzhen, China.; 10 State Key Laboratory of Natural and Biomimetic Drugs, School of Pharmaceutical Sciences, Peking University, Beijing, China.; 11 Peking-Tsinghua Center for Life Sciences, Peking University, Beijing, China.

**Keywords:** Autoimmunity, Autoimmune diseases

## Abstract

Palatine tonsils are the only air-contacted lymphoid organs that constantly engage in crosstalk with commensal microorganisms and serve as the first handling sites against microbial antigens. While tonsil inflammations have been implicated in various autoimmune diseases, including rheumatoid arthritis (RA), the precise role of tonsillar microbiota in autoimmune pathogenesis remains inadequately characterized. In this study, we profiled the tonsillar microbiota and identified a notable dysbiosis in patients with RA, particularly within the *Streptococcus* genus. Specifically, patients with RA exhibited an enrichment of pathogenic *Streptococcus* species, including *S*. *pyogenes*, *S*. *dysgalactiae*, and *S*. *agalactiae*. Colonization with these bacteria significantly exacerbated arthritis severity and increased autoimmune responses in collagen-induced arthritis (CIA). Furthermore, immunization with peptides derived from these pathogenic *Streptococcus* species directly induced experimental arthritis. Conversely, patients with RA demonstrated a marked deficiency in commensal *Streptococcus* members, notably *S*. *salivarius*. Treatment of CIA mice with *S*. *salivarius* attenuated the progression of arthritis and downregulated autoimmune responses. These findings highlight a pathogenic link of tonsillar microbiota with RA, shedding light on their contribution to autoimmunity.

## Abstract 2

The imbalance of tonsillar Streptococcus species has a significant impact on autoimmunity in rheumatoid arthritis.

## Introduction

The palatine tonsils serve as the first handling sites against microbial antigens and engage in continuous immune responses that help maintain the local and systemic immunological homeostasis (1, 2). Tonsil inflammations can exert effects on distant organs, including the heart, kidney, and joints. Tonsil-induced autoimmune/inflammatory syndrome (TIAS) is a term used to describe a group of autoimmune and inflammatory conditions characterized by chronic inflammation and immune dysregulation that originate from the tonsils. Diseases such as rheumatic fever, IgA nephropathy, and psoriasis are encompassed by this syndrome (3–5). It is hypothesized that these conditions may be triggered by the breakdown of immune tolerance to resident microorganisms within the tonsils.

Rheumatoid arthritis (RA) is an autoimmune inflammatory disorder with high morbidity and a high disability rate; it is characterized by persistent synovitis with joint destruction, systemic inflammation, and autoantibody production (6–8). The fact that RA-associated circulating autoantibodies, including rheumatoid factor and anti-citrullinated peptide antibodies, arise in the circulation and precede the onset of synovial pathology supports the notion of extra-articular origins for the autoimmune responses in RA (7). The tonsils have been implicated in the pathogenesis of RA (9–12). The tonsillar lymphocytes can migrate to subcutaneously engrafted human rheumatoid synovial tissue in mice (10), suggesting the possibility that inflammatory immune cells primed at the tonsils could migrate to the joints. Clinical data has reported that clonally identical T cell expansion occurs simultaneously in the tonsils and synovium of the same patients with RA (11, 12), lending further support to the idea of immune cell migration between tonsils and synovial tissue. However, the underlying mechanisms through which tonsils contribute to RA pathogenesis remain poorly defined.

Emerging evidence emphasizes the roles of the microbiome in the etiopathogenesis of autoimmune diseases (6–8, 13). Studies on experimental arthritis models have demonstrated that RA initiation is dependent on the participation of microorganisms, as evidenced by the fact that germ-free or antibiotics-treated mice could not develop arthritis unless particular bacteria are introduced (e.g., segmented filamentous bacteria or *Lactobacillus Bifidus* or *Prevotella copri*) (14–16). Moreover, patients with RA showed alterations in microbial compositions in multiple body sites, including the gut, the oral cavity, and the lung (17–21). The dysbiotic microbiota could trigger arthritis through activation of specific T effector cells (15, 16). Despite these advances in understanding the biological connections between the microbiome and joints, many issues remain to be addressed between the microbiome and RA pathogenesis.

There have been substantial reports linking oral microbiota to RA (17, 20–22). However, the tonsils are anatomically and functionally distinct from the oral cavity, including the tongue, gums, and teeth. The tonsils, as secondary lymphoid organs, harbor a diverse array of microorganisms that interact closely with the host immune system (2). These interactions play a crucial role in initiating immune responses against pathogens encountered through the oral and nasal routes. Research into the tonsillar microbiome has the potential to elucidate how microbial dysbiosis at this site contributes to the dysregulation of immune responses and the development of autoimmune diseases. Despite the critical role of the tonsils in immune surveillance and response, there is limited understanding of the mechanisms by which the tonsillar microbiota influence host autoimmunity. This gap in knowledge underscores the need for more comprehensive studies focusing on the microbial communities within the tonsils and their interaction with the host immune system. In the present study, we profiled the tonsillar microbiota and revealed that the tonsillar microbiota in patients with RA is characterized by a notable imbalance in *Streptococcus* species. This dysbiosis played a significant role in the development of autoimmunity, providing a mechanistic understanding that could inform future therapeutic interventions.

## Results

### Features of tonsillar microbiota in patients with RA.

To profile the tonsillar microbiota, we began with 16S rRNA gene sequencing of tonsillar swab samples from 121 patients with RA and 99 individuals acting as healthy controls (HCs) (Supplemental Table 1; supplemental material available online with this article; https://doi.org/10.1172/jci.insight.175916DS1). In addition, stool samples were collected from a subset of the cohort (*n* = 74) to delineate the relationship between the tonsillar and gut microbiota. The taxonomic compositions differed notably between the 2 habitats, with only a small fraction (4.6%) of species shared (Supplemental Figure 1, A and B). The tonsillar microbiota was characterized by a predominance of *Streptococcus*, *Prevotella*, *Fusobacteria*, and *Neisseria*, which were considerably less abundant in the gut microbiota (Supplemental Figure 1, C and D).

**Figure 1:**
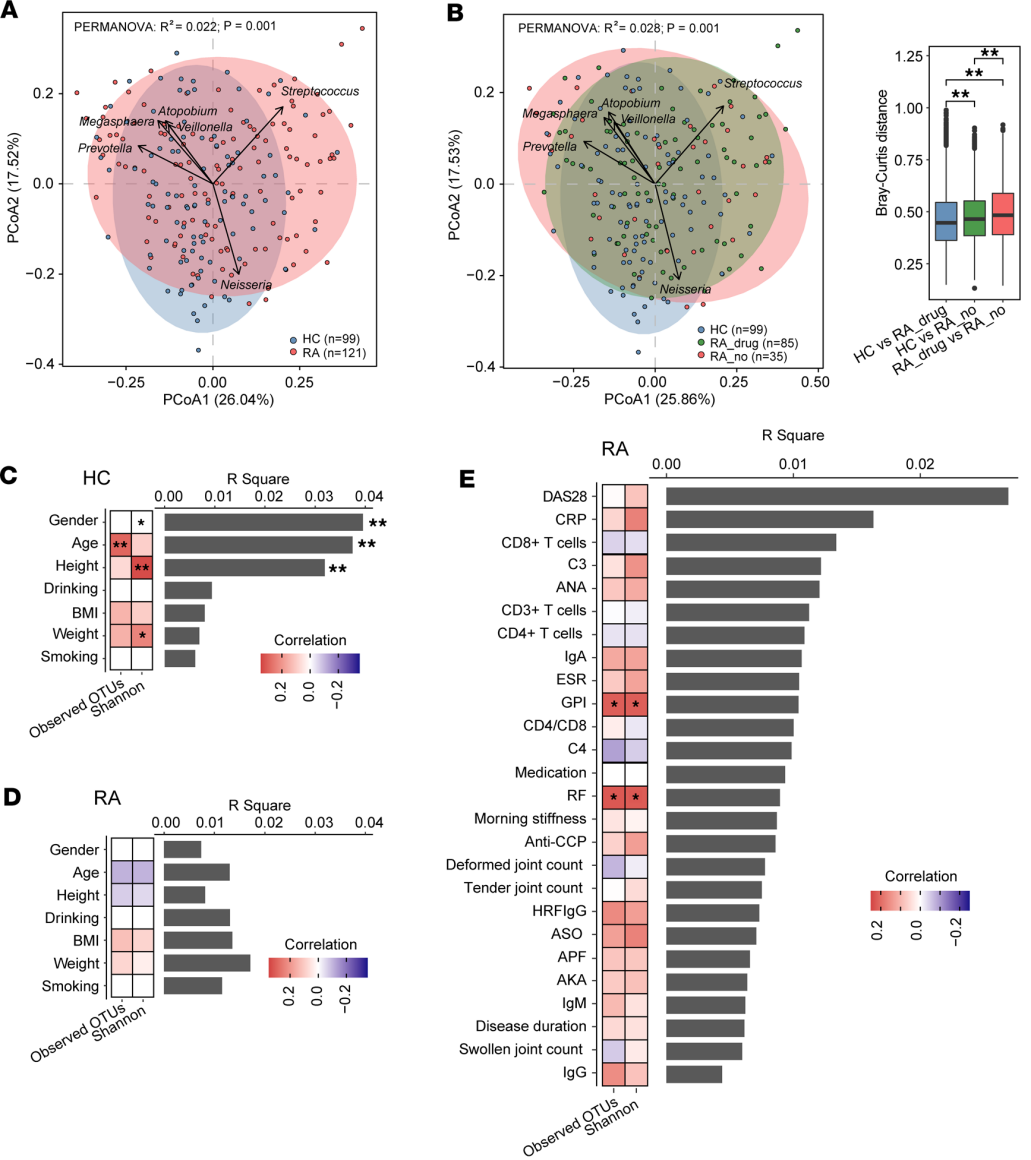
The tonsillar microbiota of patients with RA was substantially altered. (**A** and **B**) Principal coordinates analysis (PCoA), employing Bray-Curtis dissimilarity of genus-level composition, was performed. (**A**) The analysis is presented based on the first 2 axes, with the variance explained by each axis indicated in parentheses. Sample scores are depicted as points on the first 2 axes, while the scores of the top 6 genera contributing to community variation are represented as arrows. RA, rheumatoid arthritis; HC, healthy control. RA, *n* = 121; HC, *n* = 99. PERMANOVA, *P* = 0.001. (**B**) PCoA plot of the microbial compositions of individuals acting as HCs (*n* = 99); drug-treated patients with RA (*n* = 85); and patients with RA without drug treatment (*n* = 35). Analysis was performed at the genus profiles, and the top 6 genera contributing to community variation were plotted by their loadings in the PCoA plot. *P* values were calculated using Wilcoxon’s rank-sum test. **P < 0.01. (**C** and **D**) The associations of demographic factors with the interindividual variation, diversity, and richness of HC (**C**) or RA (**D**) tonsillar microbiota. (**E**) The associations of RA-associated clinical factors with the interindividual variation, diversity, and richness of tonsillar microbiota. The bar plot indicates the explained variation (R2 determined by PERMANOVA) of each factor in the interindividual variation of microbial composition (Bray-Curtis distance). The heatmaps beside the bar plot showed the correlation coefficients of each factor with Shannon’s index of diversity and observed OTUs, respectively. Statistical significance for categorical variables (age, drinking, and smoking) was determined using Wilcoxon’s rank-sum test, while Spearman’s rank correlation test was used for continuous variables (age, height, weight, and BMI). A color key for correlation is shown. *P < 0.05, **P < 0.01. The absence of an asterisk means the R² value is not significant.

The tonsillar microbiota of patients with RA showed significant increases in between-sample β-diversity, and yet it showed similar α-diversity in individuals acting as healthy controls (Supplemental Figure 2), indicating a more heterogeneous microbial community at tonsils in patients with RA. Although principal coordinates analysis (PCoA) did not reveal a clear separation between RA and control samples, permutational multivariate analysis of variance (PERMANOVA) indicated statistically significant differences in the distribution of microbial composition between the 2 groups across various taxonomic levels, even after adjusting for age, sex, and BMI (Figure 1A and Supplemental Table 2, PERMANOVA, *P* = 0.001). The tonsillar microbiota in patients with RA with medication was partially recovered to healthy conditions (Figure 1B).

Furthermore, we observed that the age, sex, and height were significantly associated with the variation of microbial composition in healthy participants, whereas these associations were almost completely diminished in patients with RA (Figure 1, C and D, and Supplemental Table 2). As the 2 cohorts differ significantly in age, we utilized PERMANOVA to explore the relationships between age and tonsillar microbial composition. Our analysis uncovered significant associations between age and microbial composition. Controlling for age reduced the effect size of disease on microbial composition, yet the statistical significance persisted (Supplemental Table 2). These results further support the fact that the tonsillar microbiota was substantially altered in disease state.

Moreover, disease activity was determined to be the top contributor to the microbial composition variation in RA tonsillar microbiota, and the autoantibodies glycosyl phosphatidyl inositol (GPI) and rheumatoid factor (RF) were significantly related with α-diversity of RA tonsillar microbiota (Figure 1E and Supplemental Table 2). Taken together, these results suggested the involvement of tonsillar microbiota in the immune responses and disease progression of RA.

### RA tonsillar microbiota was enriched by pathogenic Streptococcus species with a reduction of commensal members.

**Figure 2:**
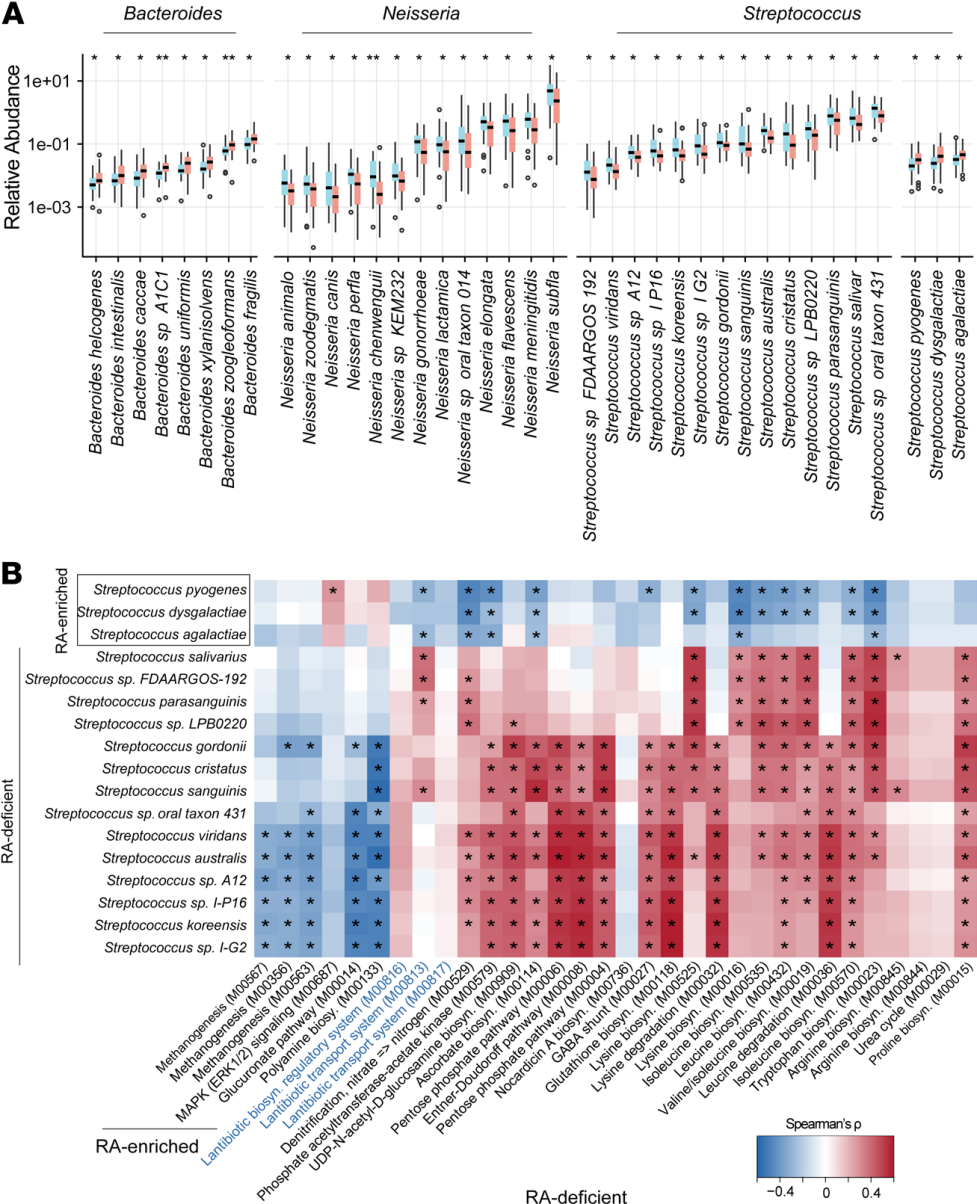
RA tonsillar microbiota is characterized by an imbalance of *Streptococcus*, *Bacteroides*, and *Neisseria* species. (**A**) Bar plots showing the differential species between patients with RA (red bars) and healthy controls (blue bars). *P* values were calculated using Wilcoxon’s rank-sum test. *P < 0.05, **P < 0.01. (**B**) Heatmap showing Spearman’s correlation coefficients between RA-associated *Streptococcus* species and the functional modules annotated using the KEGG database. Spearman’s rank correlation test, *P < 0.05.

To investigate more detailed alterations in RA tonsillar microbiome, we additionally performed whole-metagenome sequencing of tonsillar microbiota from 32 patients with RA and 30 individuals acting as healthy controls (Supplemental Table 3). Consistent with the results from 16S rRNA gene sequencing analysis, the tonsillar microbiome of patients with RA displayed a clear deviation from that of individuals acting as healthy controls, and the tonsillar microbiome in patients with medication also showed partial recovery to the healthy state (Supplemental Figure 3, A–D). A detailed comparison identified 67 species (Wilcoxon’s rank-sum test, *P* < 0.05) and 85 Kyoto Encyclopedia of Genes and Genomes (KEGG; https://www.genome.jp/kegg/) functional modules with differential abundance between patients with RA and healthy controls (Wilcoxon’s rank-sum test, FDR < 0.1) (Supplemental Tables 4 and 5). *Streptococcus* taxa comprised 25.4% (17 of 67) of the RA-associated species, followed by *Neisseria* (13 of 67, 19.4%) and *Bacteroides* (8 of 67, 11.9%) (Figure 2A, Supplemental Figure 3E, and Supplemental Table 4). Among the *Streptococcus* species, *S*. *pyogenes*, *S*. *dysgalactiae*, and *S*. *agalactiae* were enriched in RA, whereas numerous commensal *Streptococcus* species (e.g., *S*. *sanguinis*, *S*. *salivarius*, and *S*. *cristatus*) were deficient (Figure 2A). In addition, all the altered *Neisseria* members were depleted, while the *Bacteroides* species were enriched in RA (Supplemental Figure 3F), without intragenus shifts compared with that of *Streptococcus*.

Furthermore, we analyzed the correlation between RA-associated species and functional modules (Figure 2B). Since *Streptococcus* species constitute the majority of RA-related differential bacteria, we focused on these species that were depicted on the *y* axis of Figure 2B. For the modules on the *x* axis, we selected key metabolism-related modules (amino acid, carbohydrate, and energy metabolism) owing to their critical roles in microbial metabolism. Additionally, we included 3 modules related to lantibiotics because of their known function in maintaining microecological homeostasis, which is specifically relevant to RA tonsillar microbiome dysbiosis. We detected that these *Streptococcus* species showed close correlations with RA-associated functional modules (Figure 2B and Supplemental Table 5), most of which were in accordance with the observations in the gut and oral microbiota of patients with RA. By contrast, the biosynthesis and transport of lantibiotics, a series of polycyclic peptide antibiotics known to function in maintaining microecological homeostasis, were deficient in RA tonsils but not reported in the gut or oral cavity. In particular, the reduced capacity of lantibiotics biosynthesis and transport significantly associated with the deficiency of lantibiotics-producing commensal *Streptococcus* members and the enrichment of pathogenic *Streptococcus* (Figure 2B). Together, these results suggest a potential role of multiple bacterial species within the same genus *Streptococcus* in RA development.

### The imbalance of tonsillar Streptococcus species was closely associated with the immune responses of patients with RA.

**Figure 3:**
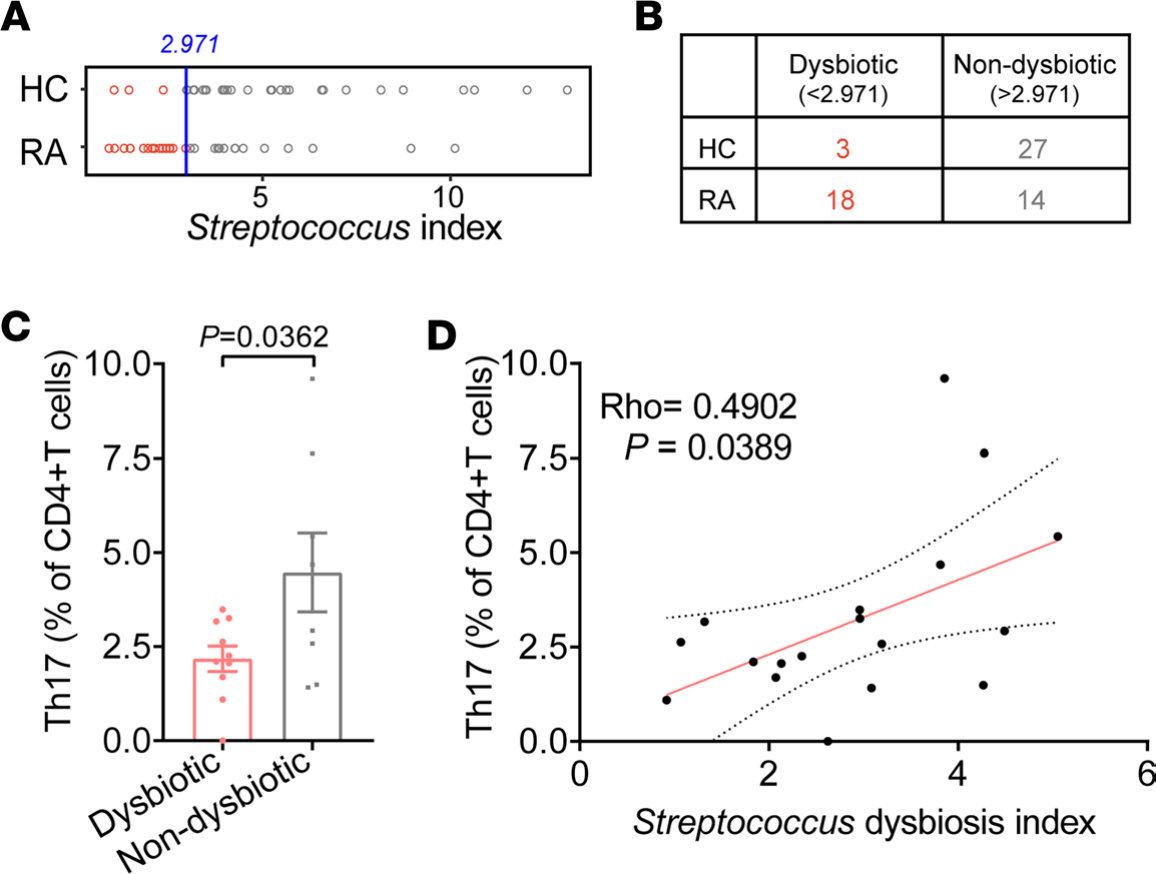
The imbalance of tonsillar *Streptococcus* species was closely associated with the immune responses of patients with RA. (**A** and **B**) The distributions of *Streptococcus* index in RA and HC tonsillar samples. Samples with *Streptococcus* index less than 2.971% were classified into the dysbiotic group. The *Streptococcus* index was defined as the value of (total abundance of *Streptococcus* members decreased in RA) minus (total abundance of *Streptococcus* species enriched in RA). The *Streptococcus* index corresponding to the best operating point was determined to be 2.971%. We consider this value as the threshold for *Streptococcus* dysbiosis. (**C**) Proportion of circulating Th17 (dysbiotic group, *n* = 10; nondysbiotic group, *n* = 8) cells in patients with RA. *P* values were calculated by using the 2-tailed *t* test. (**D**) The associations of *Streptococcus* dysbiosis index with serum levels of Th17 cells (*n* = 18). *P* values were calculated by using Spearman’s correlation test.

To comprehensively evaluate the alterations of *Streptococcus* species in tonsillar microbiota, we defined a *Streptococcus* index calculated as the difference between the total abundance of *Streptococcus* members decreased in RA and the total abundance of *Streptococcus* species enriched in RA (Supplemental Table 6). The distribution of the *Streptococcus* index is depicted in Figure 3A. Subsequently, we proposed the *Streptococcus* index as a metric for assessing the imbalance of *Streptococcus* species in the general population. The *Streptococcus* index corresponding to the optimal operating point was identified as 2.971% by using receiver operating characteristic (ROC) analysis (Supplemental Figure 4A). We regard this value as the threshold indicative of *Streptococcus* dysbiosis. Samples with *Streptococcus* index less than 2.971% were classified into the dysbiotic group (Figure 3B and Supplemental Table 6). Notably, patients with RA were significantly overrepresented in the dysbiotic group than controls (Fisher’s exact test, *P* < 0.001, Figure 3B).

Additionally, patients undergoing treatment with disease-modifying antirheumatic drugs (DMARDs), especially those receiving leflunomide or hydroxychloroquine, showed a higher propensity to appear in the nondysbiotic group compared with treatment-naive patients (Fisher’s exact test, *P* = 0.082 for DMARDs; *P* = 0.071 for leflunomide; *P* = 0.069 for hydroxychloroquine). Furthermore, we detected that Th17 cells were deficient in the *Streptococcus*-dysbiotic group of patients with RA, while no such deficiency was observed in the HC group (Figure 3C and Supplemental Figure 4, B and C). The increased *Streptococcus* index was associated with elevated Th17 cell levels in patients with RA (Figure 3D). These results indicate that intragenus dysbiosis of *Streptococcus* in the tonsillar microbiota is closely associated with host immune responses and may play a key role in RA immunopathogenesis.

### RA-enriched Streptococcus species substantially aggravated arthritis severity and increased autoimmune responses in CIA mice.

**Figure 4:**
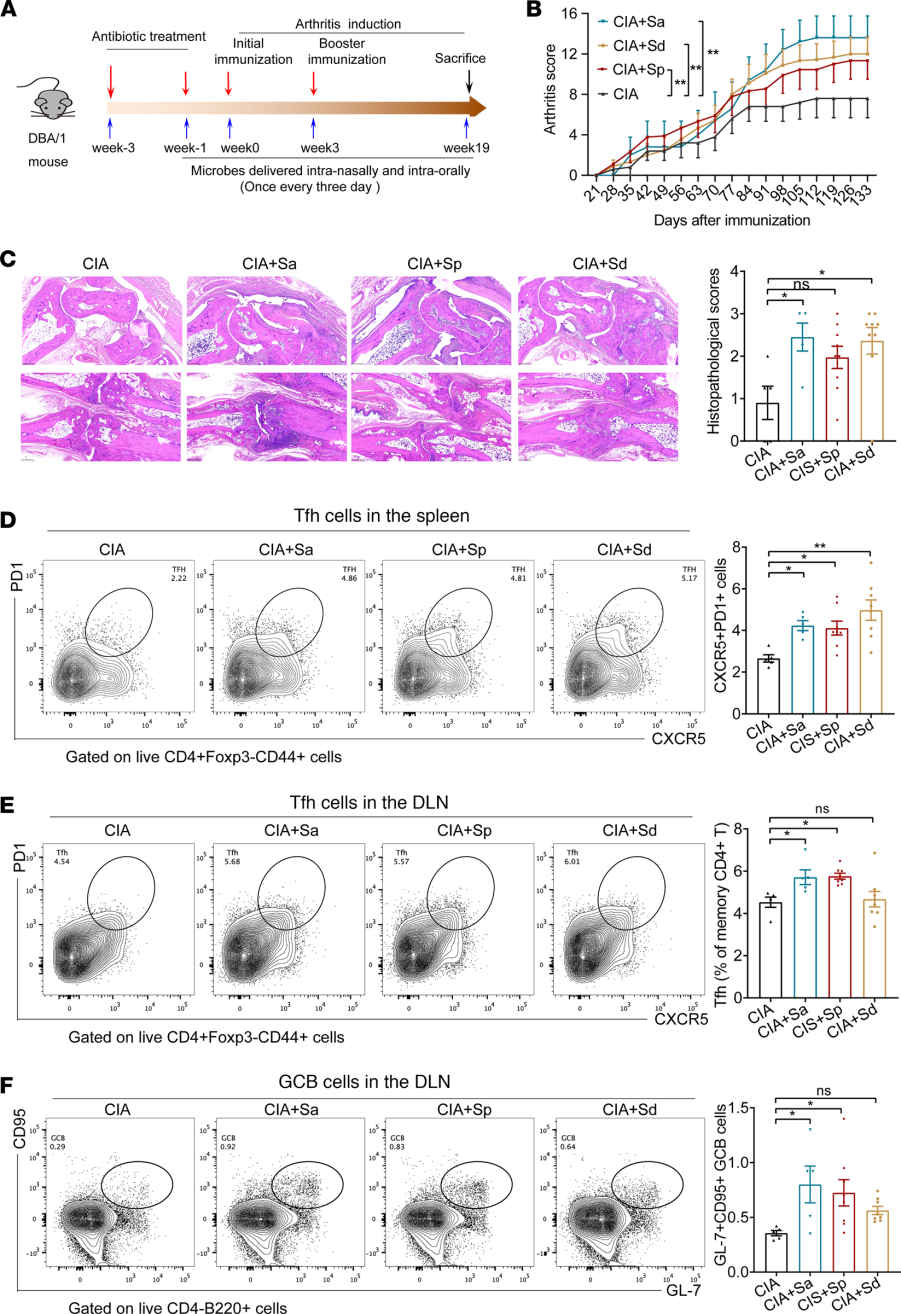
RA-enriched *Streptococcus* species significantly aggravated arthritis severity and increased autoimmune responses in CIA mice. (**A**) Schematic diagram of experimental design for intervention collagen-induced arthritis (CIA) mouse with *Streptococcus* species. (**B**) Clinical arthritis scores in CIA mice with or without *Streptococcus* species (2 × 10^8^ CFU/mice) inoculation. Sa, *S*. *agalactiae*; Sd, *S*. *dysgalactiae*; Sp, *S*. *pyogenes*. *n* = 5 for CIA. *n* = 5 for CIA+Sa, *n* = 9 for CIA+Sp, and *n* = 9 for CIA+Sd. (**C**) Representative images of H&E-stained sections and histopathological scoring of the paws in the indicated groups. *n* = 5 for CIA, *n* = 5 for CIA+Sa, *n* = 9 for CIA+Sp, and *n* = 9 for CIA+Sd. Scale bar: 250 μm. (**D**–**F**) Representative flow cytometry plots with graphs showing frequencies of Tfh (**D** and **E**, CD4^+^Foxp3^-^CD44^+^CXCR5^+^PD1^+^) and GCB (**F**, B220^+^CD4^-^Fas95^+^GL-7^+^) cells in the spleen or draining lymph modes (DLN) of indicated groups. *n* = 5 for CIA, *n* = 5 for CIA+Sa, *n* = 8 for CIA+Sp, and *n* = 8 for CIA+Sd. Data were pooled from 2 independent experiments and are expressed as mean ± SEM. Significance was determined using 2-way ANOVA followed by Tukey’s multiple comparisons test (**B**) or 1-way ANOVA with Dunnett’s multiple comparisons test (**C**–**F**). *P < 0.05, **P < 0.01.

Given the established association of *Streptococcus* species with RA, our study sought to elucidate their potential arthritogenicity in an experimental arthritis model. To this end, we administered cultures of *S*. *pyogenes*, *S*. *dysgalactiae*, or *S*. *agalactiae* strains to mice with collagen-induced arthritis (CIA), via both intraoral and intranasal routes (Figure 4A). Our findings demonstrated successful colonization of viable *Streptococcus* strains within the oropharyngeal mucosa of CIA mice (Supplemental Figure 5A). Notably, colonization with *S*. *pyogenes*, *S*. *dysgalactiae*, or *S*. *agalactiae* resulted in a significant exacerbation of arthritis severity, with no discernible effect on the onset time of arthritis (Figure 4B and Supplemental Figure 5B). Body weight was reduced in *Streptococcus*-treated mice (Supplemental Figure 5C). Histopathological evaluation of paw tissues further substantiated these observations, revealing conspicuous enhancements in synovial inflammation, bone demineralization, and cartilage deterioration among mice exposed to *S*. *pyogenes*, *S*. *dysgalactiae*, or *S*. *agalactiae* (Figure 4C).

Moreover, all 3 strains of *Streptococcus* notably augmented the frequencies of T follicular helper (Tfh) cells within both the draining lymph nodes and the spleen (Figure 4, D and E). Additionally, there was a significant increase in the frequency of germinal center B (GCB) cells within the lymph nodes (Figure 4F), indicative of heightened B cell activation and proliferation, which paralleled the observed elevation in Tfh cells. The proportions of Th1 and Th17 cells remained unaltered in mice treated with the *Streptococcus* strains (Supplemental Figure 5, D–G). Furthermore, the percentage of macrophages and monocytes showed a significant increase in *Streptococcus*-treated mice, whereas the proportions of dendritic cells and neutrophils did not display marked alterations (Supplemental Figure 5, H–K). These results demonstrated the arthritogenicity of tonsillar microbiota in RA, especially the *Streptococcus* species.

**Figure 5:**
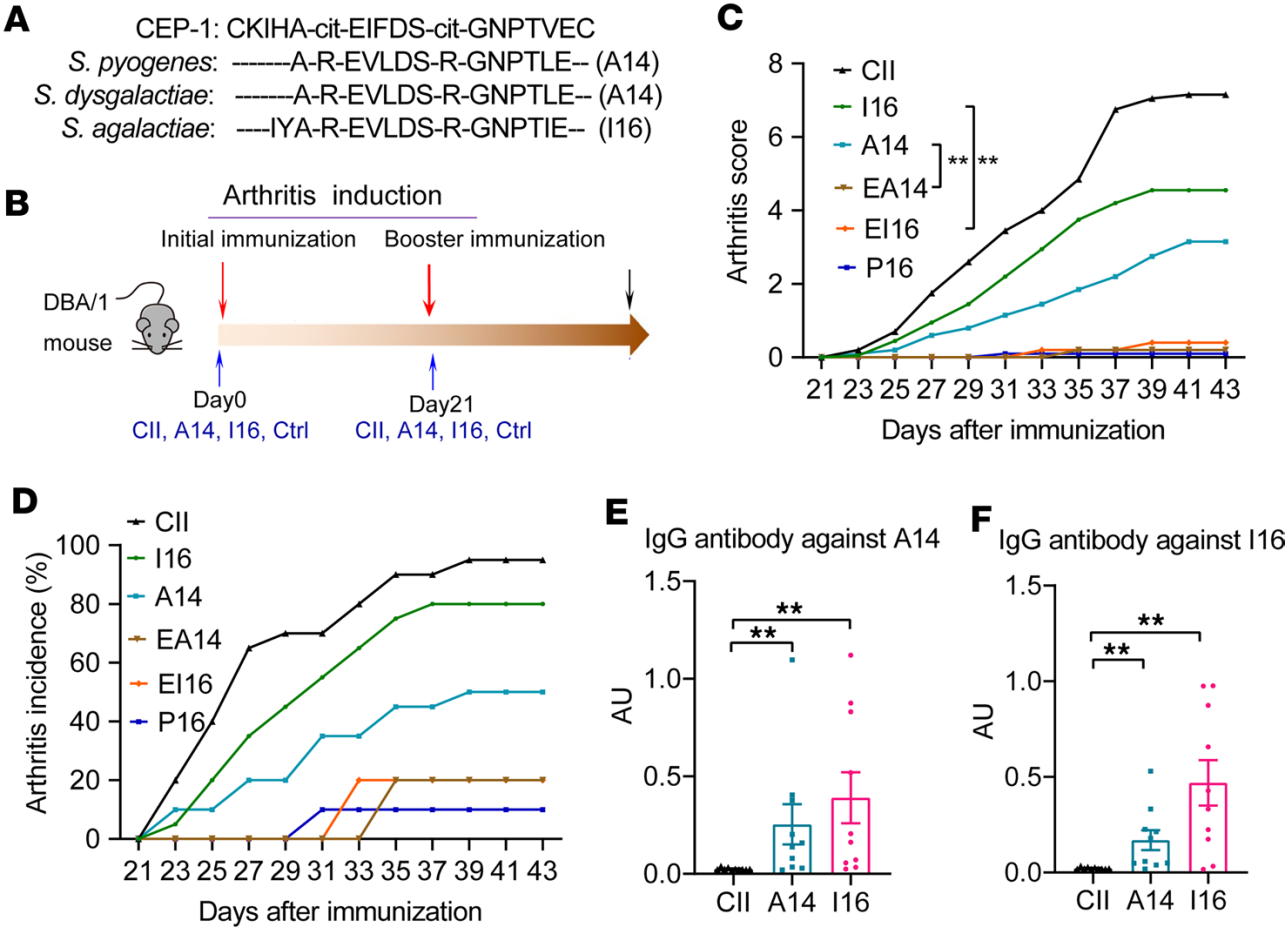
The microbial peptides derived from *Streptococcus* enolase induced experimental arthritis. (**A**) The sequences of human citrullinated α-enolase peptide 1 (CEP-1) and microbial enolase peptides from *Streptococcus*. (**B**) Schematic diagram of experimental design for inducing arthritis with collagen II (CII) or microbial peptides derived from *Streptococcus* enolase (A14 and I16). (**C** and **D**) Clinical arthritis scores and incidence in mice immunized with different peptides. *n* = 20 for CII, *n* = 20 for A14, *n* = 20 for I16, *n* = 5 for EA14, *n* = 5 for EI14, and *n* = 10 for P16. I16, IYAREVLDSRGNPTIE; A14, AREVLDSRGNPTLE; EI16, EITPNGRSDLVERAYI; EA14, ELTPNGRSDLVERA; P16, LGSMGESRRALQDSQR. (**E** and **F**) Concentrations of serum anti-IgG antibodies against A14 or I16. *n* = 10 in each group. Data were pooled from 2 independent experiments and are expressed as mean ± SEM. Significance was determined using 2-way ANOVA followed by Tukey’s multiple comparisons test (**C**) or 1-way ANOVA with Dunnett’s multiple comparisons test (**E** and **F**). **P < 0.01.

Furthermore, to determine the potential mechanism of these pathogenic *Streptococcus* species in RA, we performed a gene content analysis aiming at identifying bacterial genes containing sequences with high similarities to known RA-associated antigens. Results showed that *S*. *pyogenes*, *S*. *dysgalactiae*, and *S*. *agalactiae* all expressed enolase enzyme (VHM90835.1, MDY2963956.1, and KLL26138.1), which shared 75%–79% identities with the epitope of citrullinated α-enolase peptide 1 (CEP-1) (Figure 5A). To experimentally verify the arthritogenic roles of these microbial mimotopes, we chemically synthesized these microbial enolase-derived peptides, which were referred to as A14 (A-R-EVLDS-R-GNPTLE) and I16 (IYA-R-EVLDS-R-GNPTIE) (Figure 5A). The peptides were used to immunize DBA/1 mice, similarly to collagen II in CIA (Figure 5B). Both peptides exhibited pronounced arthritogenic effects, prompting both the initiation and advancement of arthritis, with statistical significance evident when compared with control mice immunized with scrambled peptides and the peptides from *Proteus vulgaris* (Figure 5, C and D). Moreover, both citrullinated and uncitrullinated peptides exhibited comparable efficacy in inducing arthritis (Supplemental Figure 6, A and B). Additionally, peptide CEP-1 also elicited arthritis onset in DBA/1 mice (Supplemental Figure 6, A and B). Notably, both microbial peptides elicited specific antibody responses and evoked systemic autoimmune reactions (Figure 5, E and F, and Supplemental Figure 6, C–H).

### The probiotic strain S. salivarius reduced arthritis severity and suppressed autoimmune responses in CIA mice.

Subsequently, we investigated the therapeutic potential of *S*. *salivarius* in RA by introducing *S*. *salivarius* strain K12 cultures to CIA mice. We observed successful colonization of *S*. *salivarius* K12 when administered intraorally and/or intranasally, as evidenced by its presence in the oropharyngeal mucosa of CIA mice (Supplemental Figure 7A). Additionally, a minimal colonization of *S*. *salivarius* K12 was detected in mouse lung tissues, albeit at significantly lower levels compared with the oropharyngeal mucosa (Supplemental Figure 7B).

**Figure 6:**
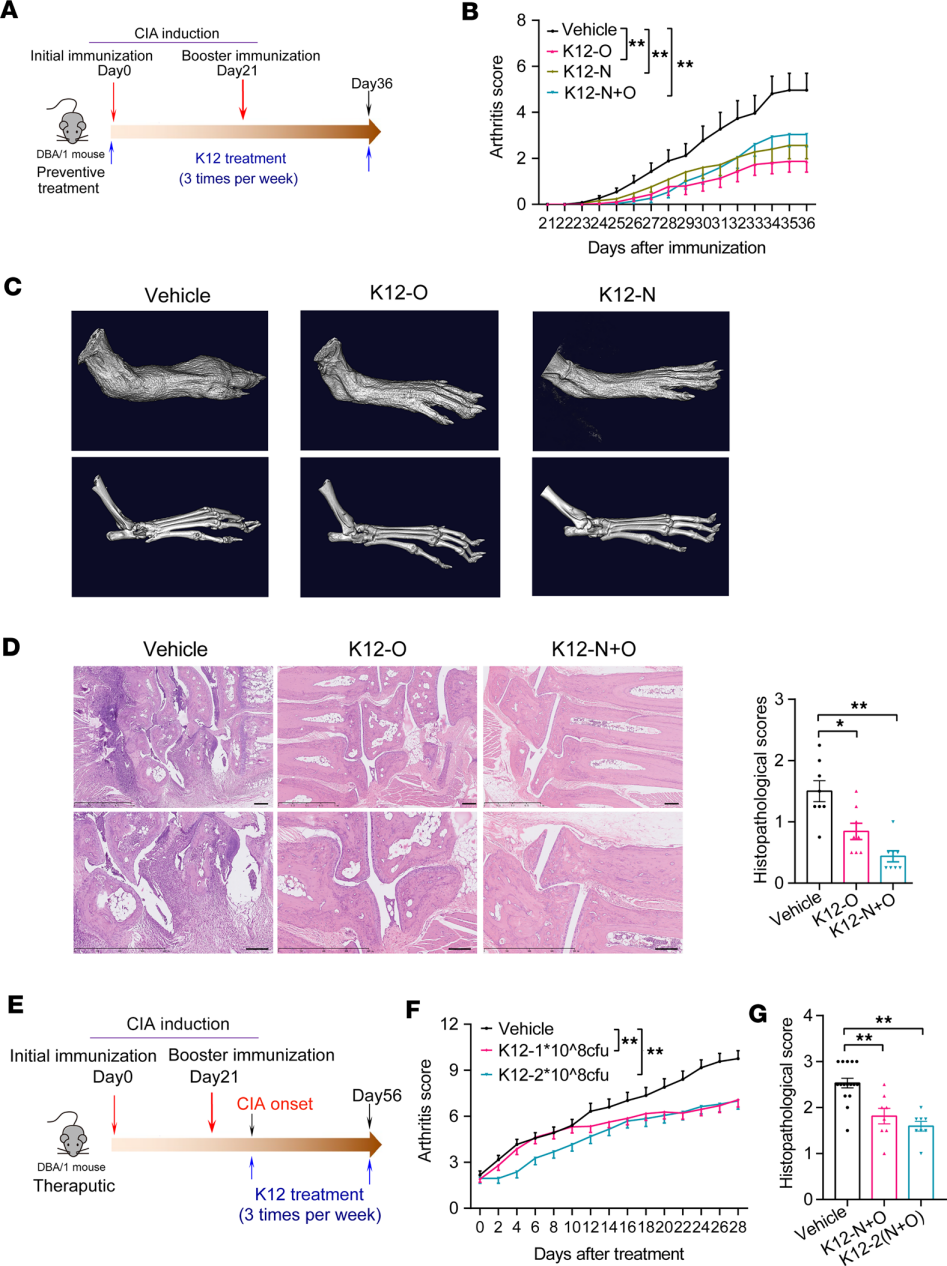
*S*. *salivarius* reduced arthritis severity in CIA mice. (**A**) Schematic diagram of experimental design for a preventive regimen with a collagen-induced arthritis (CIA) mouse model. (**B**) Clinical arthritis scores of CIA mice with or without *S*. *salivarius* K12 (1 × 10^8^ CFU/mice) inoculation. K12 strains represent distinct inoculation methods. Specifically, K12-O denotes K12 inoculated intraorally, while K12-N indicates K12 inoculated intranasally. K12-N+O indicates K12 inoculated both intraorally and intranasally. Vehicle, *n* = 26; K12-O, *n* = 25; K12-N, *n* = 30; K12-N+O, *n* = 30. (**C**) Representative images of micro-CT of the paws in the indicated groups. Scale bars: 250 mm (top), 200 μm (bottom). (**D**) Representative images of H&E-stained sections and histopathological scoring of the paws in the indicated groups. *n* = 8 per group. (**E**) Schematic diagram of experimental design for a therapeutic regimen with a CIA mouse model. (**F**) Clinical arthritis scores in CIA mice with or without *S*. *salivarius* K12 inoculation. Vehicle, *n* = 37; K12-N+O, *n* = 29; K12-2(N+O), *n* = 19. K12-1 and K12-2 represent different colonization amounts of *S*. *salivarius* K12. Specifically, K12-1 and K12-2 indicate 1 × 10^8^ CFU and 2 × 10^8^ CFU of *S*. *salivarius* K12 for each mouse, respectively. (**G**) H&E-stained evaluation of the paws in the indicated groups. Vehicle, *n* = 16; K12-N+O, *n* = 8; K12-2(N+O), *n* = 8. Data were pooled from 2 (**D** and **G**) or 3 (**B** and **F**) independent experiments and are expressed as mean ± SEM. Significance was determined using 2-way ANOVA followed by Tukey’s multiple comparisons test (**B** and **F**) or 1-way ANOVA with Dunnett’s multiple comparisons test (**D** and **G**). *P < 0.05, **P < 0.01.

In the preventative CIA models, *S*. *salivarius* K12 colonization significantly decreased the incidence and severity of arthritis without affecting body weight (Figure 6, A and B, and Supplemental Figure 8, A and B). Consistent with clinical observations, visual evaluation by micro-CT and histopathological scoring of the paws confirmed significantly reduced signs of synovial inflammation, bone destruction, and cartilage depletion in mice receiving *S*. *salivarius* K12 (Figure 6, C and D). In contrast, administration of Lactobacillus salivarius intraorally and intranasally exhibited no apparent protection from CIA (Supplemental Figure 8, C and D). Additionally, supplementation with *S*. *salivarius* K12 via intragastric gavage did not confer protection against CIA (Supplemental Figure 8, E and F).

In addition to testing its prophylactic effects, we also conducted experiments with CIA model mice that had already-developed arthritis to investigate the potential therapeutic application of *S*. *salivarius*. We found that *S*. *salivarius* K12–treated mice showed significantly relieved disease severity and reduced inflammatory cell infiltration in the joints compared with controls (Figure 6, E–G), further supporting potential clinical applicability of *S*. *salivarius* K12 in treating RA.

**Figure 7:**
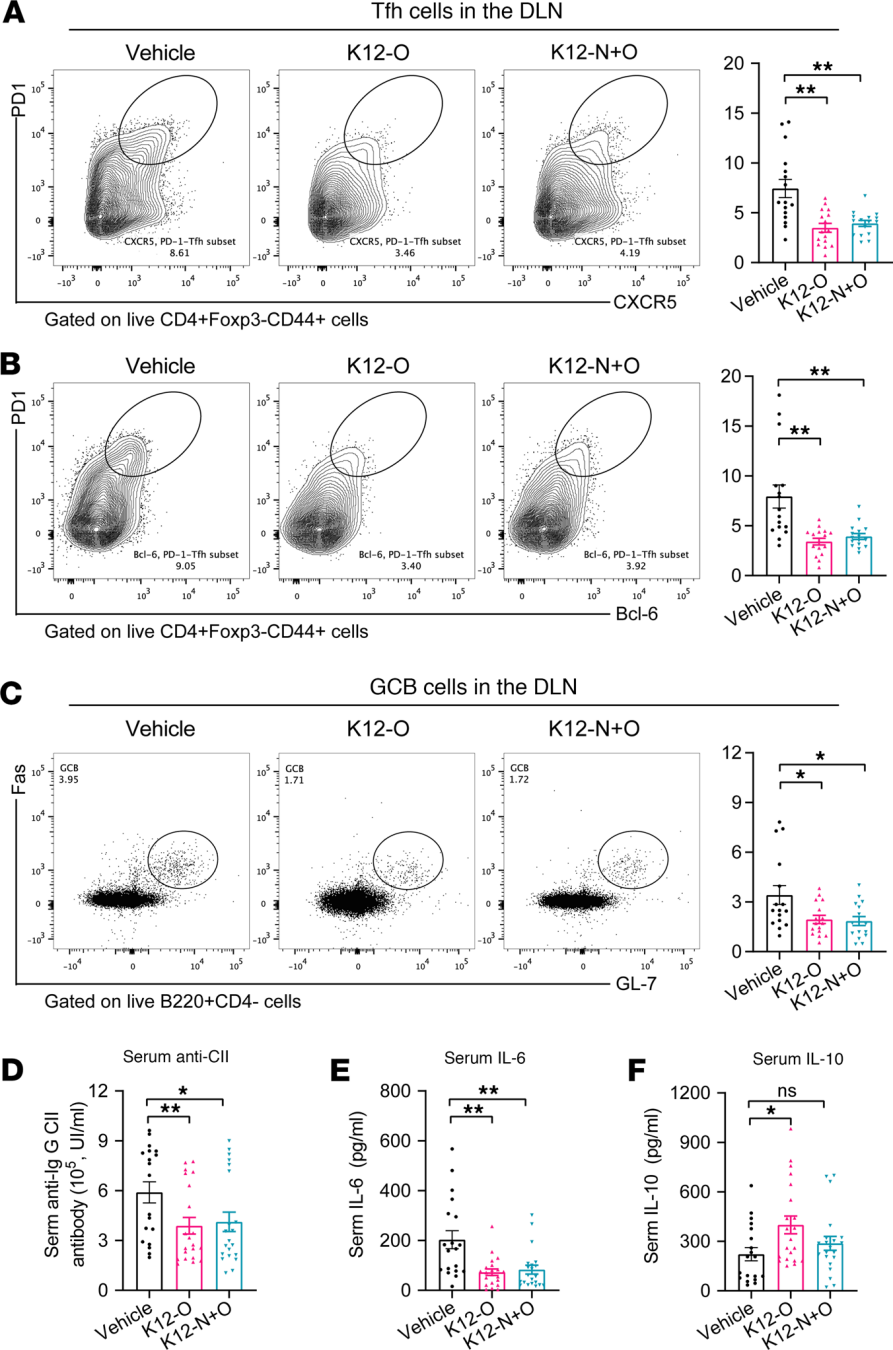
*S*. *salivarius* suppressed immune responses in experimental arthritis. (**A**–**C**) Representative flow cytometry plots with graphs showing frequencies of Tfh (**A** and **B**, CD4^+^ Foxp3^-^CD44^+^CXCR5^+^PD1^+^Bcl6^+^) and GCB (**C**, B220^+^CD4^-^Fas95^+^GL-7^+^) cells in the draining lymph modes (DLN) of indicated groups. *n* = 16 per group. (**D**–**F**) Concentrations of serum anti-collagen II (anti-CII) antibody, IL-6, and IL-10. *n* = 19 for vehicle, *n* = 21 for K12-O, and *n* = 21 for K12-N+O. Data were pooled from 3 independent experiments and are expressed as mean ± SEM. Significance was determined using 1-way ANOVA with Dunnett’s multiple comparisons test. *P < 0.05, **P < 0.01.

We next investigated which immune effector molecules may contribute to the observed antiarthritis efficacy of *S*. *salivarius* K12 by examining cytokines and lymphocyte subsets. *S*. *salivarius* K12 markedly decreased the frequencies of Tfh cells in both draining lymph nodes and the spleen (Figure 7A and Supplemental Figure 9A). Accordingly, the expression of Bcl-6, a transcription factor essential for the differentiation and function of Tfh cells, was also reduced (Figure 7B and Supplemental Figure 9A). Moreover, the frequency of GCB cells in the lymph nodes and serum autoantibody (anti-CII) level were significantly decreased (Figure 7, C and D), consistent with the downregulated Tfh cells. However, the proportions of GCB cells in the spleen were not obviously changed (Supplemental Figure 9B). Other immune cell subsets, including Th1, Th17, and Tregs, were unchanged in *S*. *salivarius* K12–treated mice (Supplemental Figure 9, C–H), suggesting selective immunomodulatory functions for *S*. *salivarius* K12 in distinct CD4^+^ T cell subsets. In addition, decreased concentration of proinflammatory cytokine IL-6 and increased serum level of antiinflammatory mediator IL-10 were detected in *S*. *salivarius* K12–treated mice (Figure 7, E and F).

More importantly, we established a functional link between *S*. *salivarius* K12 colonization and arthritis inhibition, demonstrated by the inverse correlation between *S*. *salivarius* K12 abundance and arthritis scores, as well as Tfh cell frequencies (Supplemental Figure 7, C–E). However, lung colonization of *S*. *salivarius* K12 showed no significant associations with arthritis scores or Tfh cell frequencies (Supplemental Figure 7, F–H). Furthermore, we present additional findings regarding the effect of *S*. *salivarius* K12 on the microbiome. Utilizing 16S rRNA sequencing, we observed distinct alterations in the microbial compositions within the oropharyngeal mucosa tissues of CIA mice following intraoral and/or intranasal inoculation with *S*. *salivarius* K12 (Supplemental Figure 10, A and B). Specifically, we noted a significant increase in the relative abundance of *Streptococcus* (Supplemental Figure 10C). Notably, this heightened abundance of *Streptococcus* was significantly correlated with an elevated colonization of *S*. *salivarius* K12 and reduced arthritis scores (Supplemental Figure 10, D and E). These results collectively demonstrate the active immunosuppressive effects of *S*. *salivarius* K12 in the oropharyngeal mucosa, which may be involved in inhibiting arthritis progression.

## Discussion

Focal infections localized in the tonsils have historically been implicated in autoimmune diseases, yet mechanistic elucidation has remained elusive. In this study, we described the dysbiotic composition and functional perturbations of the tonsillar microbiota, shedding light on their contributory role to disease etiology in patients with RA. Specifically, we demonstrate that an imbalance at the species level within the *Streptococcus* genus play a considerable role in the pathogenesis of this autoimmune disorder. Our findings substantially advance understanding by identifying a functional link between tonsillar microbiota and autoimmune disease, thereby providing mechanistic insights into the involvement of tonsillar bacteria in maintaining immune homeostasis.

Pathogenic species within the *Streptococcus* genus, particularly *S*. *pyogenes* and *S*. *dysgalactiae*, have long been linked to the initiation or aggravation of autoimmune disorders, including rheumatic fever and rheumatic heart disease (5, 23). Additionally, prior research has confirmed a notable association *S*. *pyogenes* and reactive arthritis (24). However, the role of *Streptococcus* species in the etiology of RA remains uncertain. Our study elucidated the involvement of *S*. *pyogenes*, along with two additional pathogenic *Streptococcus* species, in the pathophysiology of RA through the facilitation of autoimmune responses. We observed an enrichment of *S*. *pyogenes*, *S*. *dysgalactiae*, and *S*. *agalactiae* in the tonsillar microbiome of patients with RA. Notably, colonization with any of these 3 pathogenic *Streptococcus* species led to a substantial exacerbation of arthritis severity and heightened systemic autoimmune responses in murine models. Furthermore, we identified that enolase in *S*. *pyogenes*, *S*. *dysgalactiae*, and *S*. *agalactiae* exhibited substantial homology to the RA-specific autoantigen CEP-1. These enolase-derived peptides were found to trigger the onset of arthritis, induce specific antibody responses, and provoke systemic immune reactions in DBA/1 mice. These observations underscore the potential contributory role of these bacteria to the pathophysiology of RA, revealing a mechanistic pathway underlying the development of RA.

It is noteworthy that enolase exhibits high conservation and prevalence across various *Streptococcus* species. Even nonpathogenic species such as *S*. salivarius express enolase peptides that closely resemble those found in pathogenic counterparts like *S*. *pyogenes*, *S*. *dysgalactiae*, and *S*. *agalactiae*. Moreover, enolase is expressed by many other microorganisms, such as *Porphyromonas gingivalis*, which also produce enolase peptides with homology to CEP-1 (22). Therefore, the arthritogenic potential of enolase is not exclusive to the pathogenic *Streptococcus* species. The molecules and mechanisms responsible for the arthritogenicity of *S*. *pyogenes*, *S*. *dysgalactiae*, and *S*. *agalactiae* warrant further investigation to comprehensively understand their contributions to autoimmune responses and joint inflammation. Such studies are crucial for uncovering the intricate molecular pathways underlying arthritis induction by these pathogens and for developing targeted therapeutic interventions to mitigate their detrimental effects on joint health.

Moreover, these pathogenic *Streptococcus* species were observed to induce the upregulation of Tfh and GCB cells, which are pivotal in the production of autoantibodies and implicated in the pathogenesis of RA (25). Conversely, the administration of the probiotic *S*. *salivarius* was associated with a significant reduction in Tfh and GCB cell populations, resulting in the alleviation of arthritis symptoms in murine models. Our previous study has demonstrated that *S*. *salivarius*–derived antimicrobial peptides, salivaricin, can directly interact with IL-6 and IL-21 receptors (26). This interaction beneficially modulates host autoimmunity by suppressing the IL-6R/IL-21R/STAT3 signaling pathway, thereby inhibiting Tfh cell differentiation (26). These findings suggest a dynamic interplay between commensal and pathogenic *Streptococcus* species in shaping immune responses and disease outcomes. Furthermore, our study implicates the dysregulation of *Streptococcus* species as a potential driver of long-distance signaling effects, where alterations in the composition of the tonsillar microbiota exert systemic influences on autoimmune responses at distal sites. This underscores the complex and interconnected nature of the microbiome-immune axis in the pathogenesis of autoimmune diseases.

Additionally, the current study included a limited number of clinical samples to assess the imbalance of *Streptococcus* species in patients with RA. While we identified species with a *P* < 0.05 in the context of RA-associated species, it is important to note that the FDR for nearly all species was greater than 0.1. This indicates that although there are differences in abundance between the 2 groups, these differences do not reach statistical significance. Therefore, larger sample sizes are needed in future research to accurately characterize this imbalance.

Although the tonsillar microbiome exhibits distinct microbial compositions compared with the gut or other oral sites, such as the tongue, gums, and teeth (27, 28), *Streptococcus* species are found not only in the tonsils but also in the oral cavity and the gut. Our findings primarily highlight the unique microbial community within the tonsils and its association with disease outcomes. However, these results do not establish the tonsils as the exclusive site of action for these microbes. Further research, including spatial mapping of microbial communities and functional studies, is necessary to elucidate the mechanisms by which these microbes interact with the host immune system and to clarify their site-specific effects.

The tonsils are secondary lymphoid organs rich in immune cells, providing an ideal microenvironment for studying the interaction between microbial communities and the host immune system. This proximity allows for a more direct assessment of how the microbiome influences immune responses and autoimmune pathology. Studying the tonsillar microbiome allows for the identification of unique microbial signatures associated with immune-related diseases, providing insights into disease-specific microbial biomarkers and potential therapeutic targets. Manipulating the tonsillar microbiome through targeted interventions, such as probiotics, prebiotics, or microbial transplantation, holds promise for modulating immune dysregulation and alleviating autoimmune symptoms. The unique immune-privileged environment of the tonsils may enhance the efficacy of microbiome-based therapies in immune-related diseases.

In conclusion, our research highlights the key effect of the tonsillar microbiota on immune regulation and autoimmunity. The imbalance of *Streptococcus* species in the tonsils of patients with RA provides a potential perspective on the microbial contributions to autoimmune disease development and underscores the importance of maintaining a healthy tonsillar microbiome for immune homeostasis. Future studies should aim to further elucidate the specific pathways through which tonsillar microbes influence autoimmunity and explore potential microbial therapies for autoimmune conditions.

## Methods

### Sex as a biological variable.

In the human study, both male and female participants were included to ensure comprehensive representation of sex as a biological variable. In contrast, the animal study utilized only male mice; they were chosen for their reduced phenotypic variability, which helps to minimize experimental variability and improve the reliability of the results.

### Participant enrollment.

Adult patients (*n* = 121) with RA diagnosed according to the American College of Rheumatology/European League Against Rheumatism 2010 classification criteria (29) were recruited from Peking University People’s Hospital. Healthy volunteers (*n* = 99) with no history of inflammatory arthritis and rheumatic diseases were enrolled. All the participants were Chinese, and the vast majority of them were of the Han nationality. Participants were excluded if they (a) had undergone tonsillectomy; (b) had any current infection (especially in the oropharynx); (c) were suffering from serious diseases (e.g., cancer, heart failure, renal failure); (d) had taken antibiotic treatment or probiotic supplements in the 3 months prior to sample collection; (e) were strict vegetarians, alcoholics, or ate according to other nonstandard dietary modes; and (f) were pregnant or lactating women. Detailed information of the cohort was given in Supplemental Tables 1 and 3.

### Sample collection and DNA extraction.

The tonsillar microbiota samples were collected by following the procedure of Human Microbiome Project (https://hmpdacc.org/hmp/doc/HMP_MOP_Version12_0_072910.pdf). Briefly, sterile cotton swabs, premoistened in sterile saline (Beyotime), were inserted into the oral cavity and rubbed around the mucosal surface of palatine tonsil for 5 seconds without touching the uvula, tongue, or other oral structures by using of a tongue depressor. The sampling was performed twice for the left and right tonsils, respectively. The right and left sides were pooled together as a combined specimen. To detect possible contamination, several negative controls were prepared and subjected to the same procedures. Immediately after swabbing, each swab was swirled in a garnet bead tube containing 750 μL buffers (MoBio Laboratories). The swab sponge was pressed against the tube wall multiple times for 20 seconds to ensure transfer of bacteria from swab to solution. The tubes were put in a Ziploc bag and place over ice. The samples were kept at –80°C until DNA extraction.

Genomic bacterial DNA was extracted using the MoBio PowerSoil DNA Isolation Kit 12888-100 protocol (MoBio Laboratories) according to the manufacturer’s recommendations. DNA concentrations were determined using the Qubit 3.0 fluorescent quantitation kit (Thermo Fisher Scientific). Extracted DNA was stored at –80°C prior to sequencing.

### 16S rRNA gene sequencing and analyses.

The V3-V4 hypervariable regions of the 16S rRNA gene were amplified (121 patients with RA and 99 healthy controls). PCR reactions were performed by using unique fusion primers designed based on the universal primer set, 338F (5’-GTACTCCTACGGGAGGCAGCA-3’) and 806R (5’-GTGGACTACHVGGGTWTCTAAT-3’), along with barcode sequences (30). The design of the primers incorporated the Illumina adapters and a sample barcode sequence. Triplicate PCR reactions were performed for each sample and visualized on 2% agarose gels. PCR amplicons were purified using Agencourt AMPure XP kit (Beckman Coulter) and quantified with the Qubit 3.0 fluorescent quantitation kit (Thermo Fisher Scientific) and then pooled at equimolar amounts using the Illumina TruSeq Sample Preparation procedure. The amplicon library was constructed following the manufacturer’s instructions (Illumina) and quantified by KAPA Library Quantification Kit KK4824 (KAPA Biosystems). The completed library was sequenced on an Illumina MiSeq PE250 platform using dual-index sequencing strategy according to the Illumina recommended protocol (31).

16S rRNA amplicon sequences were processed based on the quantitative insights into microbial ecology platform (QIIME2, https://qiime2.org/) (32). We employed cutadapt to remove the barcode and primer sequences from both the forward and reverse reads and to truncate them at the 240th base to avoid sequencing errors at the end of the reads (33). Subsequently, flash was used to merge the forward and reverse reads, setting the minimum overlap length between two reads at 4 bp and allowing a maximum mismatch ratio of 15% (34). The merged sequences then underwent processing with the DADA2 denoise-single method to eliminate noisy sequences, chimeras, and singletons (35). The representative sequences (named “feature” in QIIME2 nomenclature) were defined at 100% similar merged sequences, after which low-occurrence (*n* < 5 in pool samples) sequences were removed. We used the term “operational taxonomic unit” (OTU) instead of “feature” herein for convenience. Then, the taxonomy of OTUs was identified using classify-sklearn classification methods based on the Greengenes v13.8 database (36) via the “q2-feature-classifier” plugin. The phylogenetic analysis was performed in QIIME2 with “qiime alignment mafft,” “qiime alignment mask,” and “qiime phylogeny fasttree” commands. Finally, all samples were rarefied to an even sampling depth of 10,000 sequences, when calculating the OTU and taxa relative abundances. To capture major changes across the whole cohort, we focused on common taxa out of all mapped ones, excluding those that were present in less than 0.01% in relative abundance.

α and β diversities were calculated in QIIME2 platform with “qiime diversity core-metrics-phylogenetic” command. The Shannon’s diversity index, observed OTUs, Faith’s phylogenetic diversity (a qualitative measure of community richness that incorporates phylogenetic relationships between the OTUs), and Pielou’s evenness were used to reflect community richness and evenness. The Jaccard distance, Bray-Curtis distance, unweighted and weighted UniFrac distances were implemented to assess the similarity or dissimilarity between individuals.

Intersample distances were calculated at the genus level using the vegdist function in R vegan, employing default parameters. PERMANOVA was then conducted using the adonis2 function from the R vegan package, based on the above distance matrix. The formula parameter used was “dist~Disease_state” where “Disease_state” indicates whether an individual is a patient with RA or a healthy control. We also computed the variation after controlling for the age variable, which is explained by the Disease_state variable, using the adonis2 function with the formula parameter “dist~Age+Disease_state”. The variation in microbial composition attributed to other variables was also computed as R² obtained from the adonis2 function, where samples with missing variables were excluded from the calculation process. PCoA was performed on the intersample distance matrix using the cmdscale function. The envfit function was used to correlate the abundance patterns of each genus with ordination axes. The scores of the top 6 genera contributing to community variation were then drawn as arrows in PCoA1 and PCoA2.

The FDR for correction of multiple comparisons was calculated using the fdrtool function with the parameter “statistic = pvalue”. *P* < 0.05 and FDR < 0.1 were considered statistically significant.

### Whole-metagenome shotgun sequencing and bioinformatic analyses.

The fresh genomics DNA samples were mechanically fragmented to approximately 400 bp with Bioruptor Pico (Diagenode, Belgium). A magnetic bead–based method was used for DNA fragments selection following a standard protocol (Agencourt AMPure XP). Libraries were prepared by using the NEBnext Ultra II DNA Library Prep Kit for Illumina (New England BioLabs). The Illumina HiSeq X platform was then used for 2 × 150 bp paired-end whole-metagenome sequencing. Initial base calling of whole-metagenome sequencing data was performed based on the system default parameters under the sequencing platform. The quality control used the following criteria: (a) reads were removed if they contained more than 3 “N” bases or more than 50 bases with low quality (<Q20); (b) no more than 10 bases with low quality (<Q20) or assigned as N in the tails of reads were trimmed. The remaining reads were then mapped to the reference human genome (GRCh38) using the Centrifuge algorithm (37) to remove host DNA contamination.

A de novo gene catalog was constructed based on the metagenomic data from the tonsillar samples of all individuals. High-quality reads were used for de novo assembly via MEGAHIT (38), which generated the initial assembly results based on different k-mer sizes and then merged and extended them using the parameter “-precorrection.” Ab initio gene identification was performed for all assembled scaffolds taking advantage of MetaGeneMark (39). The predicted genes were then clustered at the nucleotide level by CD-HIT (version 4.5.4) (40), and genes sharing greater than 90% overlap and greater than 95% identity were treated as redundancies. A nonredundant gene catalog was generated after removing the redundancy genes.

The abundance of genes in the nonredundant gene catalog was quantified as the relative abundance of reads. First, the high-quality reads from each sample were aligned against the gene catalog based on SOAP v2.21 (41) using a threshold that allowed at most 2 mismatches in the initial 32 bp seed sequence and 90% similarity over the whole read. Then, only 2 types of alignments were accepted: (a) those in which the entirety of a paired-end read could be mapped onto a gene with the correct insert size and (b) those in which one end of the paired-end read could be mapped onto the end of a gene only if the other end of the read mapped outside the genic region. The relative abundance of a given gene in a sample was finally estimated by dividing the number of reads that uniquely mapped to that gene by the length of the gene region and by the total number of reads from the sample that uniquely mapped to any gene in the catalog. The resulting set of gene relative abundances for all samples was termed a gene profile. To capture major changes across the whole cohort, we focused on common genes out of all mapped ones, excluding those that were present in less than 0.01% in relative abundance.

The KEGG database was used for functional annotation of genes using Blast KOALA (v2) (42). Each protein was assigned a KEGG ortholog (KO) on the basis of the best-hit gene (BLASTP e value < 1 × 10^–10^ and protein level similarity >30%) in the database. For KO profiling, we used KO assignment of each gene from the original gene catalog and summed the relative abundance of genes from the same KO to yield the abundance of that KO. The relative abundance of a module was calculated from summation of the relative abundance of its corresponding KOs.

### Streptococcus index.

The *Streptococcus* index was defined as the value of (total abundance of *Streptococcus* members decreased in RA) minus (total abundance of *Streptococcus* species enriched in RA). Next, we attempted to assess the performance of the *Streptococcus* index on predicting disease status (HC vs. RA) using ROC analysis. Specifically, we utilized the *Streptococcus* index of all participants as the predictor variable and their disease status as the response variable, feeding them into the roc function in the pROC package. The results demonstrated an AUC of 0.7448 for the *Streptococcus* index in predicting disease status (Supplemental Figure 3). The predictor variable (*Streptococcus* index) corresponding to the best operating point was determined to be 2.971%. We consider this value as the threshold for *Streptococcus* dysbiosis.

### Peptides synthesis.

The peptides (A14: A-R-EVLDS-R-GNPTLE; I16; EI16: EITPNGRSDLVERAYI; EA14: ELTPNGRSDLVERA; CEP-1: CKIHA-cit-EIFDS-cit-GNPTVEC; cit-I16: IYA-cit-EVLDS-cit-GNPTIE; cit-A14: A-cit-EVLDS-cit-GNPTLE) were synthesized by solid-phase techniques on a CS336X Peptide Synthesizer (Cs Bio) in RoYo Biotech Co. Ltd. We designated the peptide from *Proteus vulgaris* (43, 44) (LGSMGESRRALQDSQR) as a negative control peptide based on prior experimental evidence in CIA mice, where it showed a negative effect on arthritis induction (Jing Li, unpublished data). The peptides were purified by high-performance liquid chromatography (Shimadzu Corp.) with a purity of more than 97% and identified by a Liquid chromatography-mass spectrometer (Shimadzu Corp.).

### Bacterial culture.

Under sterile conditions, inoculate bacterial strains were added into corresponding liquid culture media at a ratio of 0.1% and incubated at 37°C with agitation at ~44.72 g for 1 day. *S*. *pyogenes* (ATCC, 19615) and *S*. *dysgalactiae* (ATCC, 35666) were cultured in brain heart infusion broth. *S*. *agalactiae* (ATCC, 13813) was cultured in tryptic soy broth supplemented with 5% defibrinated sheep blood. *S*. *salivarius* K12 (ATCC, BAA1024) and *Lactobacillus salivarius* (ATCC, 11741) were cultured in Todd-Hewitt broth. Genomic DNA was extracted from 200 μL of cultured bacterial liquid using the proteinase K digestion method, and the remaining bacterial liquid was stored at 4°C. Full-length 16S sequencing was conducted on the bacterial strains, BLASTN alignment was performed against the NCBI database, and the top hit was selected as the species information for the sample. A small amount of bacterial liquid was diluted in PBS buffer at a gradient, with a 10^4^-fold dilution as the first gradient, followed by 3-fold dilutions, for a total of 6 gradients. 100 μL of each gradient of bacterial liquid was evenly spread onto culture plates and incubated at 37°C for 2 days. The single bacterial colonies were counted on the plates, and the bacterial concentration in the original bacterial liquid was calculated.

### Experimental arthritis induction.

Male DBA/1 mice (6–8 weeks old) were purchased from Huafukang Co. Ltd. and fed under specific pathogen–free conditions. Experimental arthritis induction was established in the same way as CIA by following a previously published protocol (45). Briefly, DBA/1 mice were immunized intradermally at the base of the tail with 200 μg bovine type II collagen (CII, Chondrex) or different peptides (A14, I16, EI16, EA14, CEP-1, cit-I16, cit-A14) emulsified in complete Freund’s adjuvant (Sigma-Aldrich) in equal volumes. Three weeks later, a booster was delivered using 100 μg CII or different microbial peptides emulsified in Freund’s incomplete adjuvant (Sigma-Aldrich). Mice were monitored weekly for weight and for signs of arthritis following the booster immunization. Clinical score was assessed by using the following system as reported previously (45): 0, normal; 1, erythema and swelling of 1 or several digits; 2, erythema and moderate swelling extending from the ankle to the midfoot (tarsals); 3, erythema and severe swelling extending from the ankle to the metatarsal joints; and 4, complete erythema and swelling encompassing the ankle, foot, and digits, resulting in deformity and/or ankyloses. The scores of all 4 limbs were summed, yielding total scores of 0–16 per mouse.

### Experimental arthritis intervention.

Mice were randomized into treatment groups. After antibiotic treatment (0.5 mg/mL ampicillin sodium, neomycin sulfate, and metronidazole and 0.25 mg/mL Vancomycin HCl in the drinking water) for 2 weeks, *S*. *dysgalactiae* (ATCC, 35666), *S*. *agalactiae* (ATCC, 13813), or *S*. *pyogenes* (ATCC, 19615) was inoculated intranasally and orally at a dose of 2 × 10^8^ CFU per mouse, and administered once every 3 days starting 1 week prior to the initial immunization. *S*. *salivarius* K12 was administered 3 times a week via the following routes: intranasally (10^8^ CFU per mouse), orally (10^8^ CFU per mouse), combined intranasally and orally (10^8^ CFU per mouse), or intragastrically (10^9^ CFU per mouse). The preventive group started on day 1, and the therapeutic groups commenced after the onset of CIA (approximately day 26). *Lactobacillus salivarius* (ATCC, 11741) inoculated intranasally and intraorally (1 × 10^8^ CFU/mice) 3 times a week starting from the initial immunization. Mice treated with sterile PBS (Thermo Fisher Scientific) served as controls. All the antibiotics were bought from Macklin Co. Ltd.

### Determination of Streptococcus strains colonization.

Under sterile conditions, samples of oropharyngeal mucosal tissue were thoroughly homogenized. The samples were diluted in PBS buffer at a 10-fold gradient, creating 4 gradients in total. 100 μL of each diluted sample was evenly spread onto culture plates. The plates were incubated on selective streptococcal culture medium at 37°C under aerobic conditions for 1 day. Based on the culture results, plates with an appropriate number of single bacterial colonies were selected. The number of single colonies grown on each plate was counted, and bacterial cells were picked for genomic extraction using the proteinase K digestion method, with up to 96 single colonies picked per sample. Following Illumina high-throughput sequencing requirements, bidirectional sequencing was performed and fusion primers with “5′ adapter-barcode-sequencing primer-specific primer-3’” were designed. Libraries were constructed using a 2-step PCR amplification method. Based on the colony count from culture plates and high-throughput sequencing results (for sequencing results, after removal of the added quantities of control sequences, which validated the sequencing process, if the abundance of *Streptococcus* in the sample was above 30%, it was considered as a *Streptococcus* colony), the concentration of *Streptococcus* in the original samples was calculated.

### Determination of S. salivarius K12 abundance.

Genomic DNA was extracted from the oropharyngeal mucosa using a Protease K Fasting Burst Technique. The DNA concentrations were evaluated spectrophotometrically. The quantity of *S*. *salivarius* K12 in the samples was estimated by quantitative real-time PCR (qPCR) with SRBR Premix Ex Taq (2×) (Takara) and FTC-3000TM Real-Time Quantitative Thermal Cycler (Funglyn). All qPCR reactions were run with 3 replicates per DNA. Standard curves were set up by serially diluting plasmid of a pMD18-T vector (Takara) with the appropriate insert from 10^7^ to 10^2^ target K12 gene copies/μL. The standard curve was obtained using linear regression of threshold cycle numbers (C_T_) versus log copy numbers of targets. *S*. *salivarius* K12–specific primers were as follows: forward, 5′-AAGGGAGAATGATTGCCATGAA-3′; reverse, 5′-GAGTTTGGACAGTCATCAGTAATAGTTG-3′. As TaqMan probe, K12Taq was designed as follows: 6-FAM-5′-AGAGGTACAGGTTGGTTTG-3′-MGB (46). qPCR reactions were performed in a 25 μL reaction volume containing 12.5 μL SRBR Premix Ex Taq (2×) (Takara), 1 μL (10 μM) each of the forward and reverse primer, 5 μL DNA, 2 μL probe, and 3.5 μL of RNAse/DNAse-free PCR water. PCR reaction conditions included an initial denaturation at 94°C for 10 minutes, followed by 45 cycles of 94°C for 1 minute and 60°C for 105 seconds. Melting curve analyses were performed from 60°C to 96°C with increments of 0.1°C per cycle. The gene copy number in each sample was determined by comparing to Ct values/gene copy number of the standard curve.

### Radiography evaluations and histological analyses.

The paws from each mouse were fixed in 4% paraformaldehyde and then scanned using a Micro-CT scanner (Quantum FX, Caliper). After that, the paws were decalcified in 5% EDTA, paraffin-embedded, sectioned, and stained with H&E. A microscopic assessment of sagittal sections was performed, and histopathological changes were scored based on the previously reported parameters (47): 0, normal synovium; 1, synovial membrane hypertrophy and cell infiltrates; 2, pannus and cartilage erosion; 3, major erosion of cartilage and subchondral bone; and 4, loss of joint integrity and ankylosis. The scores of all 4 limbs were summed and divided by 4, yielding average scores of 0–4 per mouse.

### Cytokine and autoantibody detection.

At the study endpoints, mice were euthanized and serum samples were collected for cytokine and autoantibody detection. The concentrations of cytokines (IL-6 and IL-10) were measured using ELISA kits (Multisciences), and the titer of collagen-specific antibody was analyzed using a mouse anti-bovine type II collagen IgG antibody assay kit (Chondrex). Serum levels of anti-*Streptococcus* enolase peptides were tested by indirect ELISA. In detail, chemosynthetic peptides (10 μg/mL in 0.05 M carbonate buffer) were coated in the 96-well plates (Corning) at 4˚C overnight. Then, the 96 wells were washed with PBS plus 0.05% Tween-20 (PBST) 3 times before blocking them with 5% BSA-PBST at 37˚C for 3 hours. All serum samples were diluted at 1:100 with 1% BSA-PBST, and 100 μL diluted samples were added to the 96-well plate and incubated 2 hours at 37˚C. 1% BSA-PBST was used as control nonspecific background. After incubation, the wells were washed 4 times with 0.05% PBST. Then, 100 μL of goat anti-mouse IgG conjugated to peroxidase (1:10,000 in 1% BSA-PBST) was added to the wells and incubated at 37˚C for 40 minutes. The bound antibodies were tested by tetramethylbenzidine as substrate after washing 4 times. This reaction was terminated by adding 50 μL of 2 M sulfuric acid to the wells. The absorbance density (OD) was read by a Bio-Rad plate reader at 450 nm and 630 nm. The OD values were converted into AU values, which was calculated as follows: AU = [(OD peptide – OD nonspecific background) test serum/(OD peptide – OD nonspecific background) positive control serum] × 100.

### Flow cytometry.

The spleens and joint draining lymph nodes (popliteal and axillary lymph nodes) were obtained from mice, sieved through a 70 μm cell strainer (Corning) in RPMI 1640 medium with 10% FBS, and single-cell suspensions (10^6^ cells/100 μL) were prepared for flow cytometry. For intracellular cytokine analysis, cells were stimulated with 25 ng/mL PMA and 1 μg/mL ionomycin plus 10 μg /mL brefeldin A (PeproTech) for 4 hours. Cell surface markers were stained first, and the cells were then fixed and permeabilized with an intracellular staining buffer set (Thermo Fisher Scientific) following the manufacturer’s protocol and stained with intracellular or intranuclear markers. Antibodies were purchased from eBiosciences (Thermo Fisher Scientific), BD Biosciences, or Life Technologies (see Supplemental Table 7). Th1 cells were CD4^+^IFN-γ^+^, Th17 cells were CD4^+^IL-17A^+^, Tregs were CD4^+^CD25^+^FOXP3^+^, Tfh cells were CD4^+^Foxp3^–^CD44^+^CXCR5^+^PD-1^+^Bcl6^+^, and GCB cells were B220^+^CD4^–^Fas95^+^GL-7^+^. Detailed information on the antibodies is given in Supplemental Table 7. Flow cytometry was performed using FACSAria II (BD Biosciences), and the data were analyzed using FlowJo v10.0.7 software (Tree Star Inc.).

### Statistics.

The statistical analyses for microbiome were performed using the R v3.3 platform (https://www.r-project.org/). PERMANOVA was performed with the R *vegan* package, and the *P* value was generated based on 999 permutations. Distance-based redundancy analysis was performed on these taxonomic composition profiles with the *vegan* package, based on the Bray-Curtis dissimilarly, and visualized via the R *ade4* package. The RA-associated species and modules were identified based on the Wilcoxon’s rank-sum test. The *q* value was used to evaluate the FDR for correction of multiple comparisons and was calculated based on the R *fdrtool* package. *P* < 0.05 and *q* < 0.1 was considered statistically significant. Correlation analysis was carried out by using the Spearman’s rank correlation statistical measurement system (48). One patient with RA was excluded from the clinical association analyses due to the missing information.

For the experiments, the differences of arthritis scores and incidence between groups were determined by 2-way ANOVA followed by Tukey’s multiple comparisons test and Kaplan-Meier analysis with log-rank test, respectively. The differences of cytokines, antibodies, lymphocytes, or DNA abundance between 2 groups were analyzed by Mann-Whitney *U* nonparametric test. The correlation between variables was evaluated using the Spearman’s rank correlation test. Statistical analyses were performed by the GraphPad prism v8.00 software. A 2-sided *P* value of less than 0.05 was considered statistically significant.

### Study approval.

Informed consent was obtained from all participants. This study was approved by Peking University People’s Hospital Ethics Committee. All experiments were carried out in accordance with guidelines prescribed by the Animal Care and Use Committee of Peking University People’s Hospital.

### Data availability.

The raw sequencing dataset acquired in this study has been deposited to the China National GeneBank (CNGB) database under accessions CNP0002433 (https://db.cngb.org/search/?q=CNP0002433) and CNP0002434 (https://db.cngb.org/search/?q=CNP0002434). The authors declare that all other data supporting the findings of this study are available within the article. Supporting data values are available from the corresponding authors upon reasonable request. Values for all data points in graphs are reported in the Supporting Data Values file.

## Author contributions

ZL, JW, and JL conceived and designed the project. JL, JJ, Y Jin, Y Zhong, HY, YA, and Y Jia were in charge of participant enrollment and sample collection. RY and Y Zhou carried out DNA extraction, PCR, and microbiota sequencing. SL, RG, Y Zhou, XZ, and BX completed all the bioinformatic and statistical analyses of the microbiota. JL, JJ, LC, XC, PR, Y Jin, XX, and WX performed all the in vitro and in vivo experiments. JL, SL, JJ, RG, XS, FH, and JG acquired and processed data as well as conducted figure preparations. JL, SL, and JJ wrote the manuscript with input and edits from ZL, JW, JH, JQ, and LW. All the authors have revised and approved the manuscript submission.

## Supplementary Material

Supplemental data

Supplemental tables 1-7

Supporting data values

## Version 1 08/20/2024

In-Press Preview

## Version 2 09/24/2024

Electronic publication
