# Supplementary material for: The aberrant tonsillar microbiota modulates autoimmune responses in rheumatoid arthritis
Source: JCI Insight. 2024 Aug 20;9(18):e175916. doi: 10.1172/jci.insight.175916 (PMC11457857; doi:10.1172/jci.insight.175916)
Supplement: Supplemental data [file jciinsight-9-175916-s199.pdf]

Supplementary Materials for

The aberrant tonsillar microbiota modulates autoimmune responses in rheumatoid arthritis

Jing Li[1,2†], Shenghui Li[3,4†], Jiayang Jin[1,2†], Ruochun Guo[4], Yuebo Jin[1,2], Lulu Cao[1,2], Xuanlin Cai[1,2], Peishi Rao[1,2], Yan Zhong[1,2,5], Xiaohong Xiang[1,2], Xiaolin Sun[1,2], Jianping Guo[1,2], Fanlei Hu[1,2], Hua Ye[1,2], Yuan Jia[1,2],Wenjing Xiao[6], Yuan An[1,2], Xuan Zhang[7,8], BinBin Xia[7,8], Rentao Yang[9], Yuanjie Zhou[9], Lijun Wu[5], Junjie Qin[9], Jing He[1,2]*, Jun Wang[7,8]*, Zhanguo Li[1,2,10,11]*

[1]Department of Rheumatology and Immunology, Peking University People's Hospital, Beijing, 100044, China

[2]Beijing Key Laboratory for Rheumatism Mechanism and Immune Diagnosis (BZ0135), Beijing, 100044, China

[3]Key Laboratory of Precision Nutrition and Food Quality, Department of Nutrition and Health, China Agricultural University, Beijing, 100083, China

[4]Puensum Genetech Institute, Wuhan 430076, China

[5]Department of Rheumatology and Immunology, The People's Hospital of Xin Jiang Uygur Autonomous Region, Urumqi, 830001, China.

[6]Emergency Department, Peking University People's Hospital, Beijing, 100044, China

[7]CAS Key Laboratory for Pathogenic Microbiology and Immunology, Institute of Microbiology, Chinese Academy of Sciences, Beijing, 100101, China

[8]University of Chinese Academy of Sciences, Beijing, 100049, China

[9]Promegene Translational Research Institute, Shenzhen, 518110, China

[10]State Key Laboratory of Natural and Biomimetic Drugs, School of Pharmaceutical Sciences, Peking University, Beijing, 100191, China

[11]Peking-Tsinghua Center for Life Sciences, Peking University, Beijing, Beijing, 100091, China

*** Corresponding authors**

Prof. Zhanguo Li, Department of Rheumatology and Immunology, Peking University People's Hosptial, No.11, Xizhimen South Street, Xicheng District, Beijing, China; Phone number: +86-13910713924; E-mail: li99@bjmu.edu.cn.

Prof. Jun Wang, CAS Key Laboratory for Pathogenic Microbiology and Immunology, Institute of Microbiology, Chinese Academy of Sciences, No.1, Beichen West Road, Chaoyang District, Beijing, China; Phone number: +86-18354305753; E-mail: junwang@im.ac.cn.

Prof. Jing He, Department of Rheumatology and Immunology, Peking University People's Hosptial, No.11, Xizhimen South Street, Xicheng District, Beijing, China; Phone number: +86-18611707374; Email: hejing1105@126.com.

†These authors contributed equally to this work.

This file includes:

Figs. S1 to S10

SFig. 1

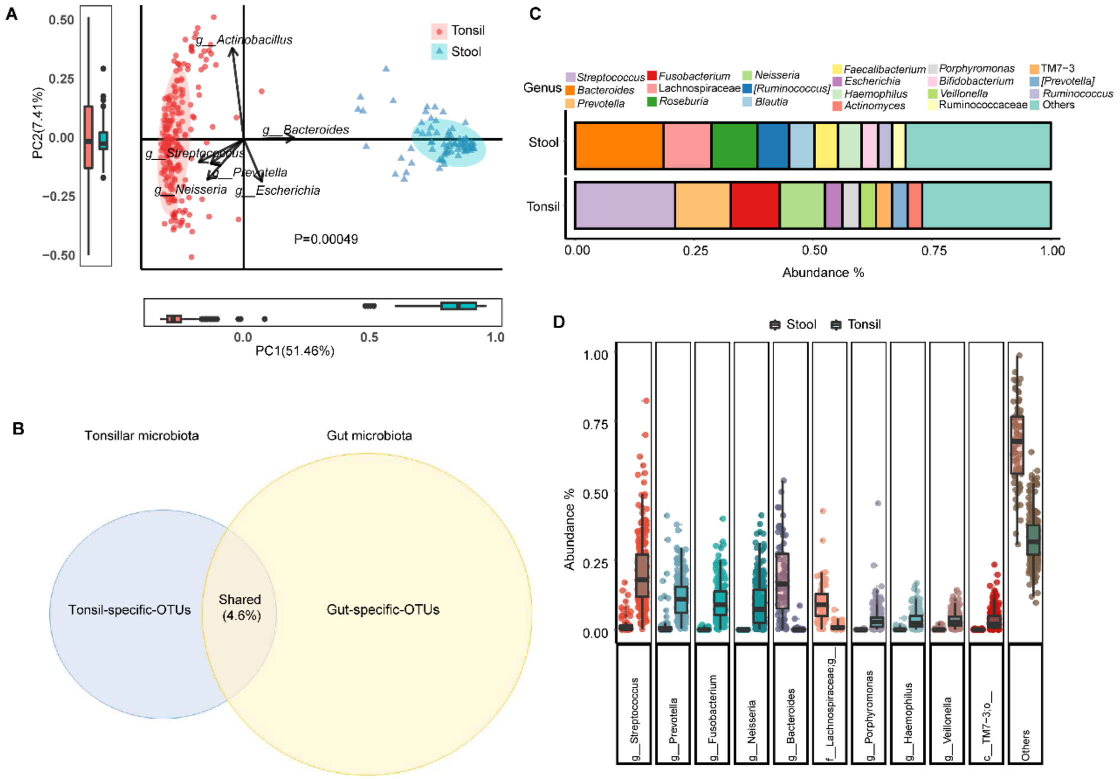

Supplementary Figure 1. The tonsillar microbiota was quite distinct from gut microbiota (A) Principal component analysis (PCA) plots based on the genus profiles revealed that the tonsillar samples (red dots, n= 220) clustered separately from the stool samples (blue dots, n=74) (Permutational multivariate analysis of variance, PERMANOVA, P = 0.00049). PC: principal component. **(B)** The compositions of microbiota in the tonsils and gut. In the 74 pairs tonsil and gut microbiota samples, only a tiny fraction of OTUs (4.6%) were tonsil-gut-shared. **(C and D)** Bar plots of the top 10 most abundant genera in the tonsil and gut microbiota. Each dot represented one sample. n=74.

SFig. 2

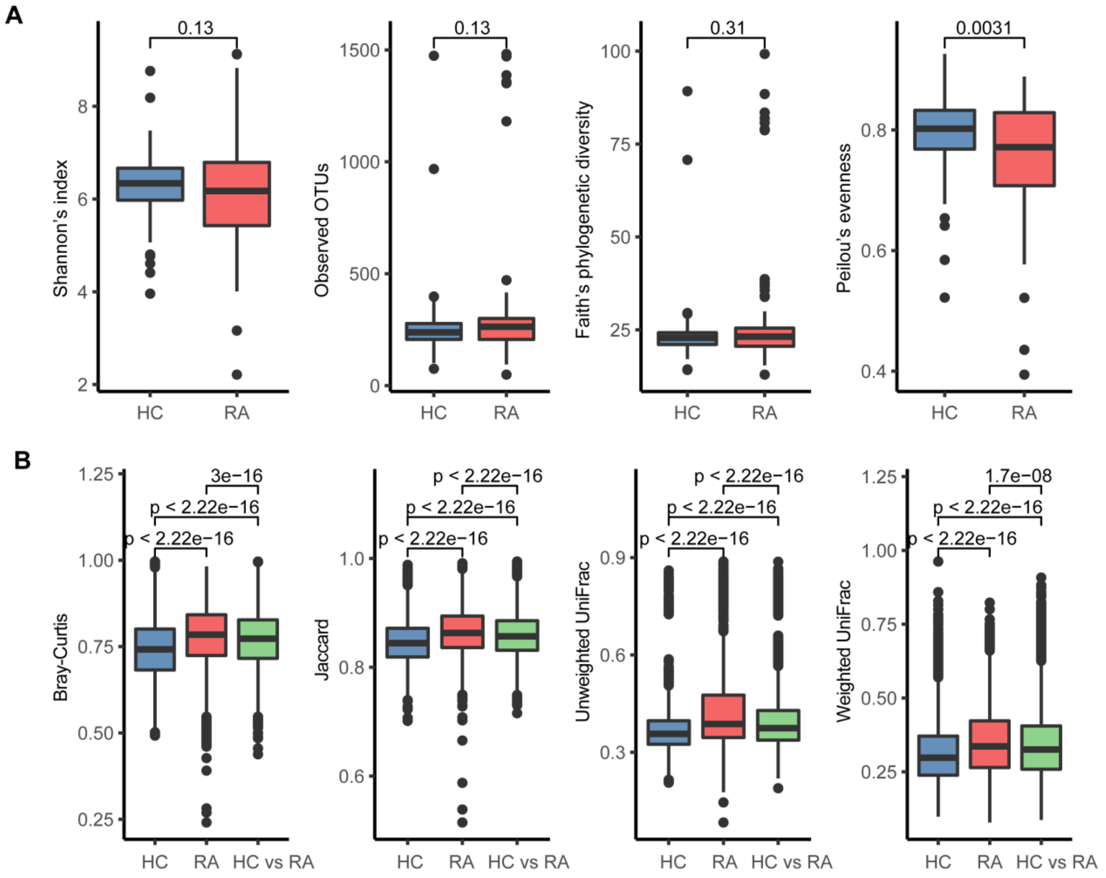

Supplementary Figure 2. The alternations of microbial diversity in RA tonsillar microbiota. (**A** and **B**) Comparison of α (A) and β (B) diversity indexes of tonsillar microbiota between healthy controls (HC) and rheumatoid arthritis (RA) patients. Boxes depicted the interquartile ranges (IQRs) between the first and third quartiles, and the line inside the box denoted the median; whiskers represented the lowest or highest values within 1.5 times IQR from the first or third quartiles. Dots represented data point beyond the whiskers. RA, n=121; HC, n=99. P-values were calculated using Spearman's rank correlation test.

SFig.3

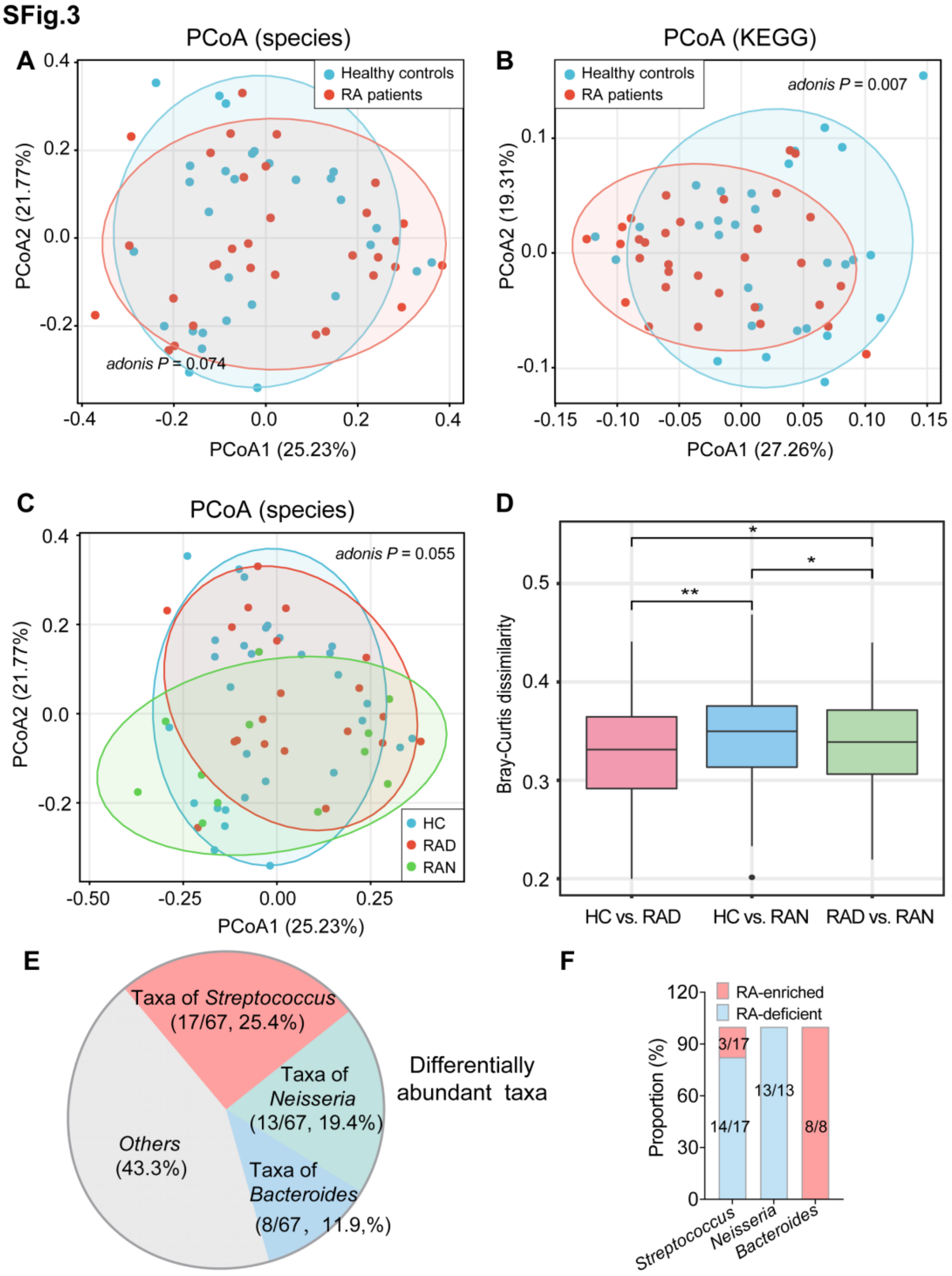

Supplementary Figure 3. The tonsillar microbiome is significantly altered in RA patients. (**A** and **B**) The tonsillar microbiota of RA patients (n=32) and healthy controls (n=30) were profiled by using a standard whole-metagenome shotgun sequencing technology. dbRDA of Bray-Curtis dissimilarity revealed a clear deviation of the tonsillar microbiota of RA patients from healthy controls at the microbial species level (a, Adonis, P < 0.001, R2= 0.12564) and the KEGG ortholog level (b, Adonis, P = 0.009, R2 = 0.04878). Samples were shown at the primary constrained axis (CAP1) and primary multidimensional scaling axis (MDS1), and lines connected samples in the same group. (**C**) Distance-based redundancy analysis (dbRDA) of Bray-Curtis dissimilarity among the microbial compositions of healthy controls (HC, n = 30), RA patients with drug treatment (RAD, n = 20) and without drugs (RAN, n =

12). Samples were shown at the primary constrained axis (CAP1) and primary multidimensional scaling axis (MDS1), and lines connected samples in the same group. (**D**) Bray-Curits dissimilarly analysis among healthy controls, RA patients with drug treatment (RAD) or without drugs (RAN). Analysis was performed at the species profiles, *P* values were calculated using Wilcoxon rank-sum test. $*P < 0.05$, $**P < 0.01$. (**E**) Pie diagram showing the differentially abundant taxa between RA patients and healthy controls. (**F**) The proportions of bacteria that enriched or deficient in RA among *Streptococcus*, *Neisseria*, and *Bacteroide*s.

SFig. 4

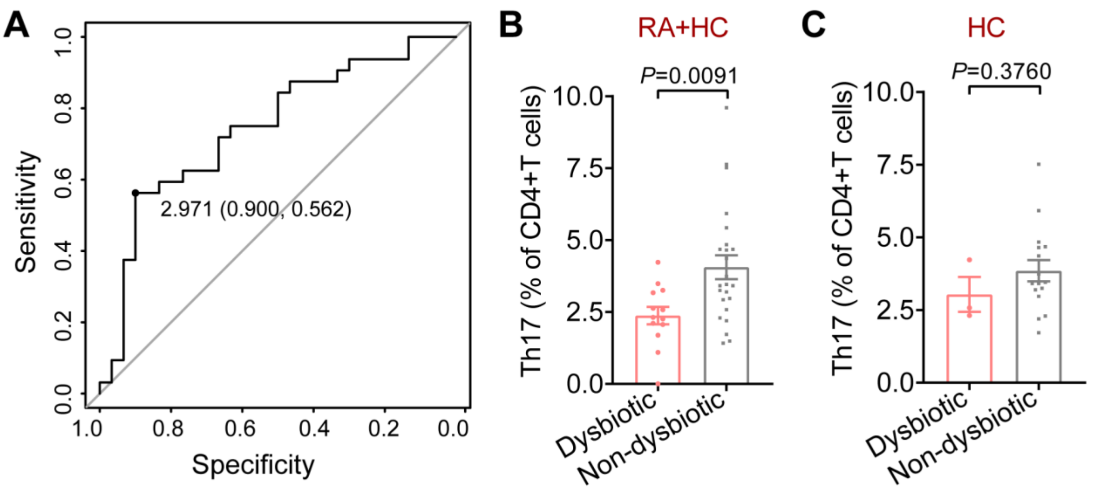

Supplementary Figure 4. Intra-genus dysbiosis of *Streptococcus* in tonsillar microbiota. (**A**) ROC analysis identified 2.971% as the threshold for *Streptococcus* dysbiosis. **(B)** Proportions of circulating T helper 17 (Th17, dysbiotic group, n=13, non-dysbiotic group, n=24) cells in RA patients and HC group. **(C)** Proportions of circulating T helper 17 (Th17, dysbiotic group, n=3, non-dysbiotic group, n=16) cells in HC group. P-values were calculated by using t test.

SFig. 5

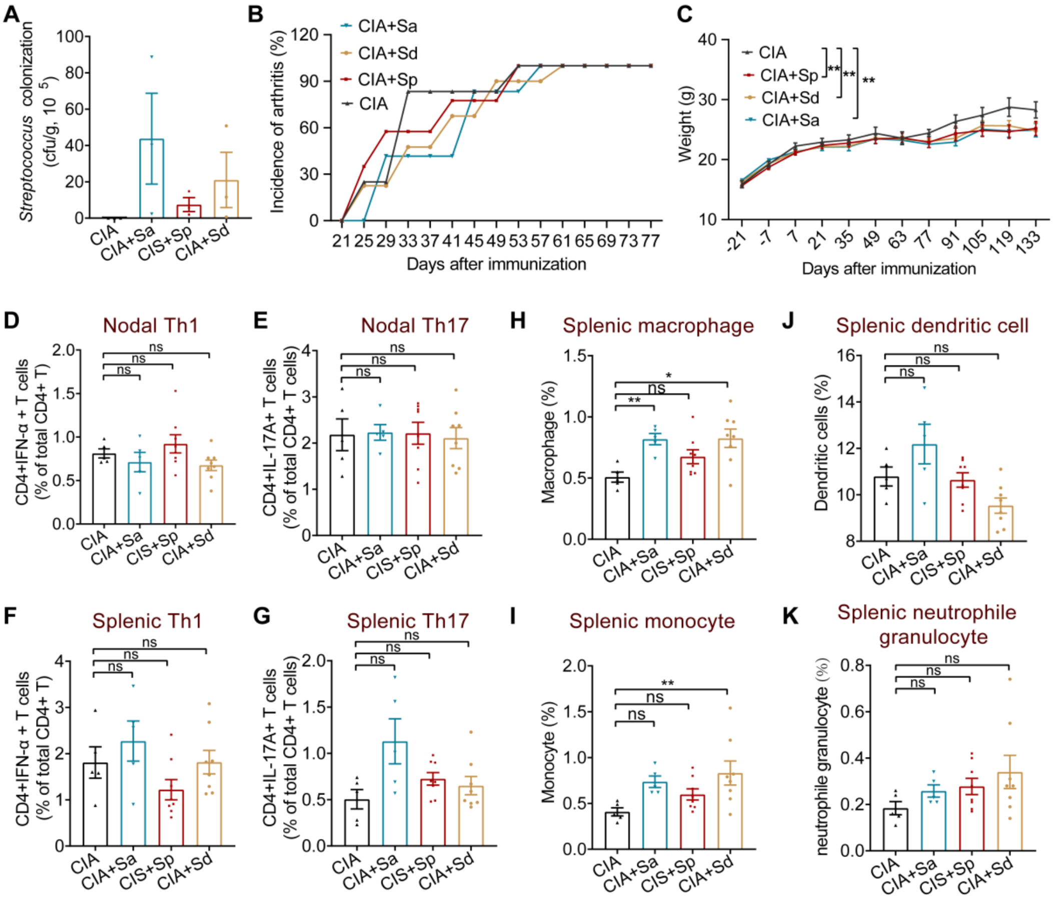

Supplementary Figure 5. The effect of pathogenic *Streptococcus* species in CIA mice. (A) The colonization amount of *Streptococcus* strains in the oropharyngeal mucosa of CIA mice. Sa indicates *S. agalactiae*. Sd indicates *S. dysgalactiae*. Sp indicates *S. pyogenes*. n=3 in each group. **(B and C)** Arthritis incidence (B) and body weight (C) in CIA mice with or without *Streptococcus* strains (2*10^8cfu per mice) inoculation. n=5 for CIA, n=5 for CIA+Sa, n=9 for CIA+Sp, and n=9 for CIA+Sd. (**D-G**) Proportions of Th1 and Th17 cells in the draining lymph modes or the spleen. n=5 for CIA, n=5 for CIA+Sa, n=8 for CIA+Sp, and n=8 for CIA+Sd. (**H-K**) The percentages of macrophages, monocytes, dendritic cells, and neutrophils in the spleen. n=5 for CIA, n=5 for CIA+Sa, n=8 for CIA+Sp, and n=8 for CIA+Sd. Data were pooled from two independent experiments (B-H) and are expressed as the mean ± SEM. Significance determined using two-way ANOVA followed by Tukey's multiple comparisons test (C), or Mann-Whitney U test (D-K). *$P < 0.05$, **$P < 0.01$, ns: non-significant.

SFig. 6

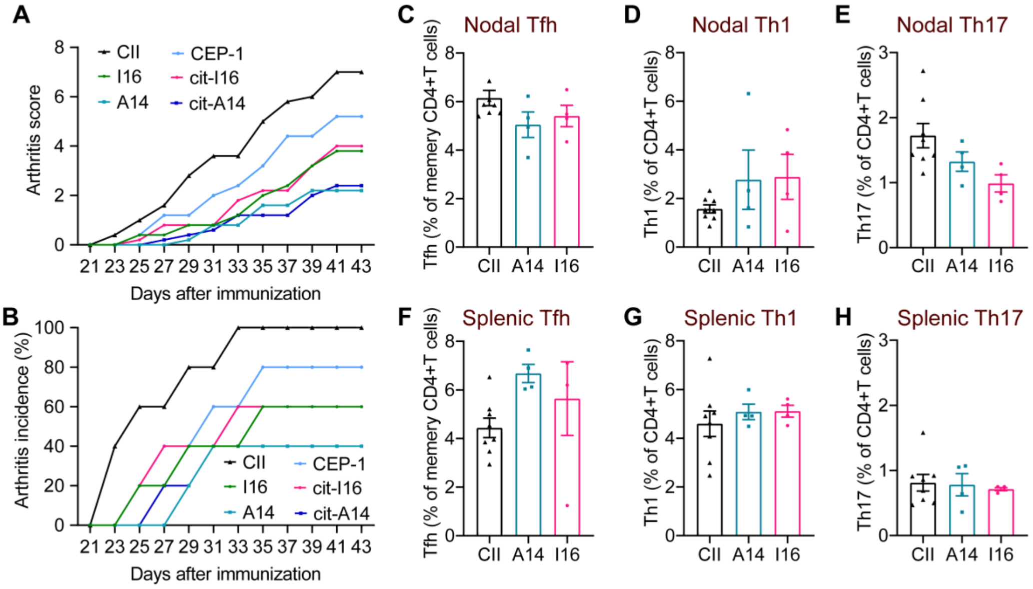

Supplementary Figure 6. The microbial peptides derived from *Streptococcus* enolase induced experimental arthritis. (**A** and **B**) Clinical arthritis scores and incidence in mice immunized with different peptides. cit-I16: IYA-cit-EVLDS-cit-GNPTIE; cit-A14: A-cit-EVLDS-cit-GNPTLE; CEP-1: CKIHA-cit-EIFDS-cit-GNPTVEC. n=5 in each group. (**C-H**) Proportions of Tfh, Th1 and Th17 cells in the draining lymph modes or the spleen. n=8 for CII, n=4 for A14 and I16. Data were expressed as mean ± SEM.

SFig. 7

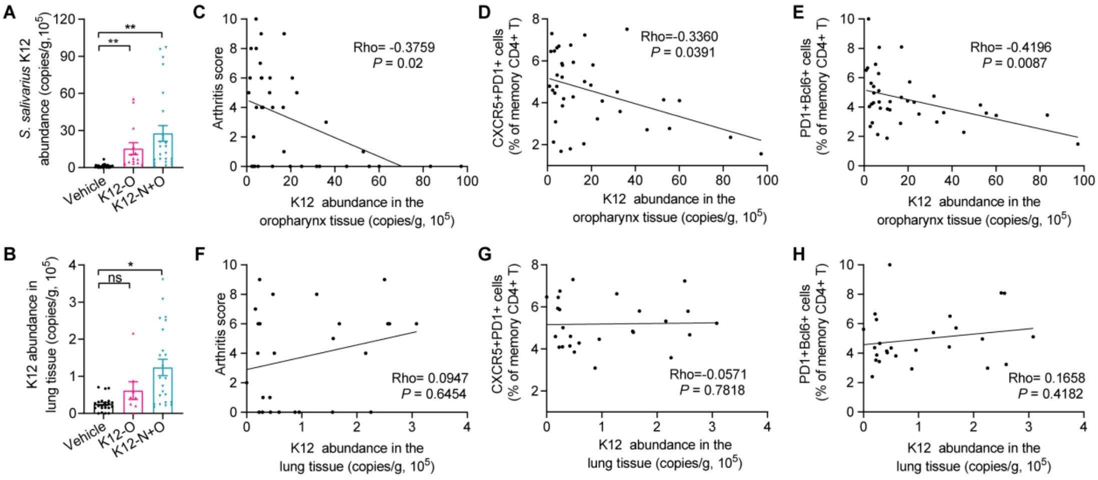

Supplementary Figure 7 The colonization of *S. salivarius* K12 and its associations with arthritis scores and Tfh cell frequencies. (A) Quantitative real-time PCR (qPCR) indicating the abundance of *S. salivarius* K12 in the oropharyngeal mucosa of CIA mice. Vehicle, n=31; K12-O indicates S. salivarius K12 administered intra-orally, n=14. K12-N+O indicates *S. salivarius* K12 administered intra-nasally and intra-orally, n=25. **(B)** qPCR indicating the abundance of *S. salivarius* K12 in the lung tissues of CIA mice. Vehicle, n=22; K12-O, n=8; K12-N+O, n=23. **(C-E)** The scatter plots depict associations of *S. salivarius* K12 abundance in the oropharyngeal mucosa of mice with arthritis scores or Tfh cell frequencies, n=38. **(F-H)** The scatter plots depict associations of *S. salivarius* K12 abundance in the lung tissue of mice with arthritis scores or Tfh cell frequencies, n=26. Data were pooled from two independent experiments and are expressed as the mean ± SEM. Significance was determined using Kruskal-Wallis test followed by Dunn's multiple comparisons test (A and B) or Spearman's rank test (C-H). $^*P < 0.05$, $^{**}P < 0.01$, ns: non-significant.

SFig.8

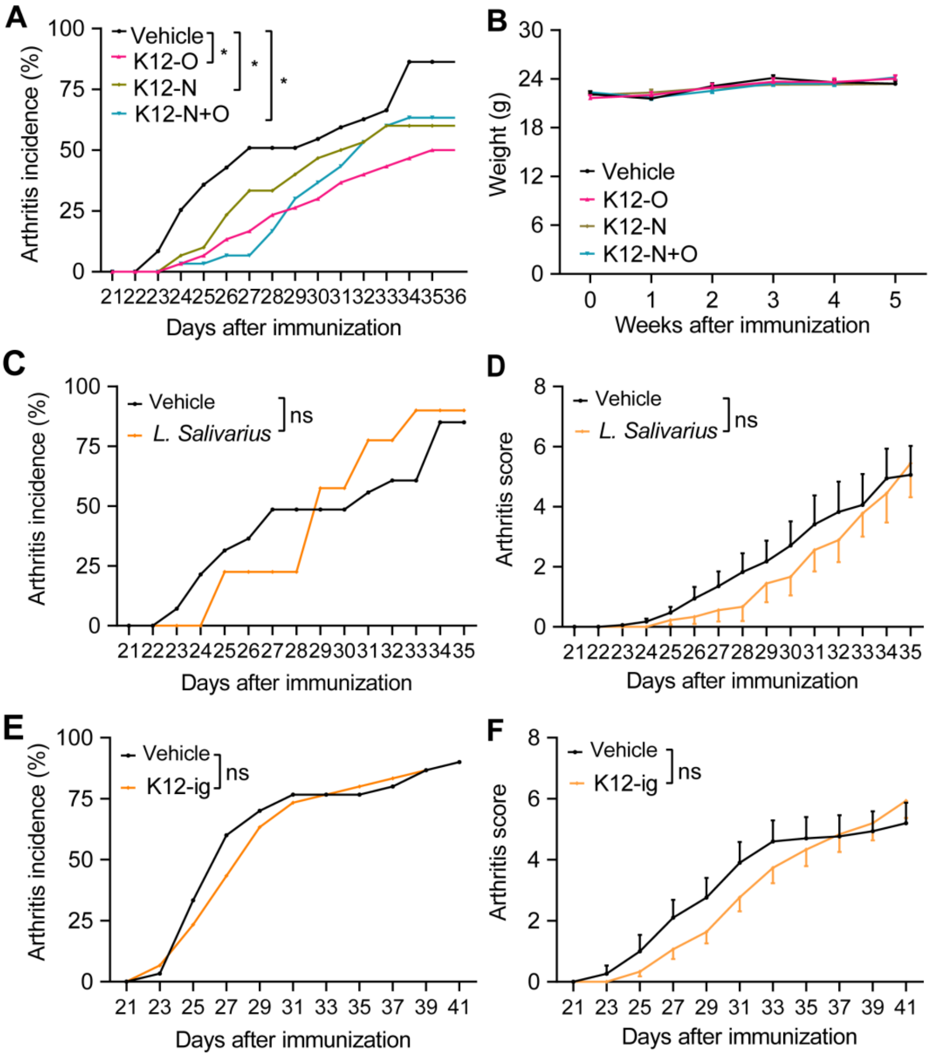

Supplementary Figure 8. Inoculation with *L. Salivarius* intra-orally and intra-nasally *or S. salivarius* intragastrically showed no protection from arthritis in CIA mice. (**A and B**) Arthritis incidence (A) and body weight (B) in CIA mice with or without *S. salivarius* K12 ($1*10^8$cfu/mice) inoculation. K12 strains represent distinct inoculation routes. Specifically, K12-O denotes K12 inoculated intra-orally, while K12-N indicates K12 inoculated intra-nasally. K12-N+O indicate K12 inoculated both intra-orally and intra-nasally. For A, Vehicle, n=26; K12-O, n=25; K12-N, n=30; K12-N+O, n=30. For A and B, n=16 for Vehicle, n=20 for other groups. (**C** and **D**) Incidence and clinical scores of arthritis in CIA mice with or without *L. Salivarius* ($1*10^8$cfu/mice) treatment intra-orally and intra-nasally. Vehicle, n=17; *L. Salivarius*: *Lactobacillus salivarius,* n=9. (**E** and **F**) Incidence and clinical scores of arthritis in CIA mice with or without *S. salivarius* K12 ($1*10^9$cfu/mice) inoculation. n=30 per group. ig: intragastric administration. Data were pooled from two (B, C, D) or three (A, E, F) independent experiments and

expressed as mean ± SEM. Significance determined using Kaplan-Meier analysis with log-rank test (A, C, and E) or two-way ANOVA followed by Tukey's multiple comparisons test (D and F). ns: non-significant.

SFig.9

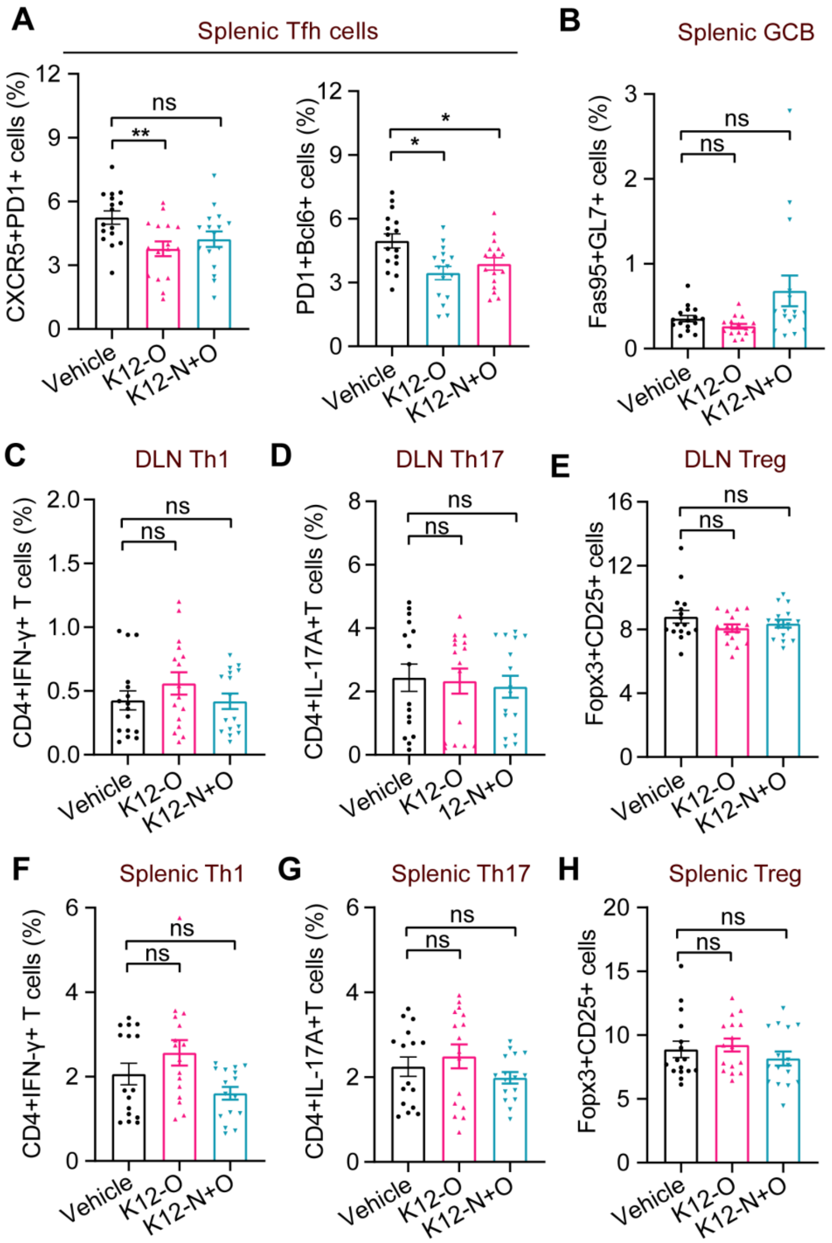

Supplementary Figure 9. *S. salivarius* suppresses immune responses in experimental arthritis. (**A-H**) Graphs of frequencies of Tfh (A, CD4$^+$CD44$^+$CXCR5$^+$PD1$^+$Bcl6$^+$), GCB (B, B220$^+$CD4$^-$Fas95$^+$GL-7$^+$), Th1 (C and F, CD4$^+$IFN-γ$^+$), Th17 (D and G, CD4$^+$IL-17A$^+$), and Treg (E and H, CD4$^+$CD25$^+$Fopx3$^+$) cells in the spleen of indicated mice. n=16 per group. Data were pooled from two independent experiments and expressed as mean ± SEM. Significance determined using One-way ANOVA with Dunnett's multiple comparisons test (A-H). *$P < 0.05$, **$P < 0.01$, ns: non-significant.

SFig. 10

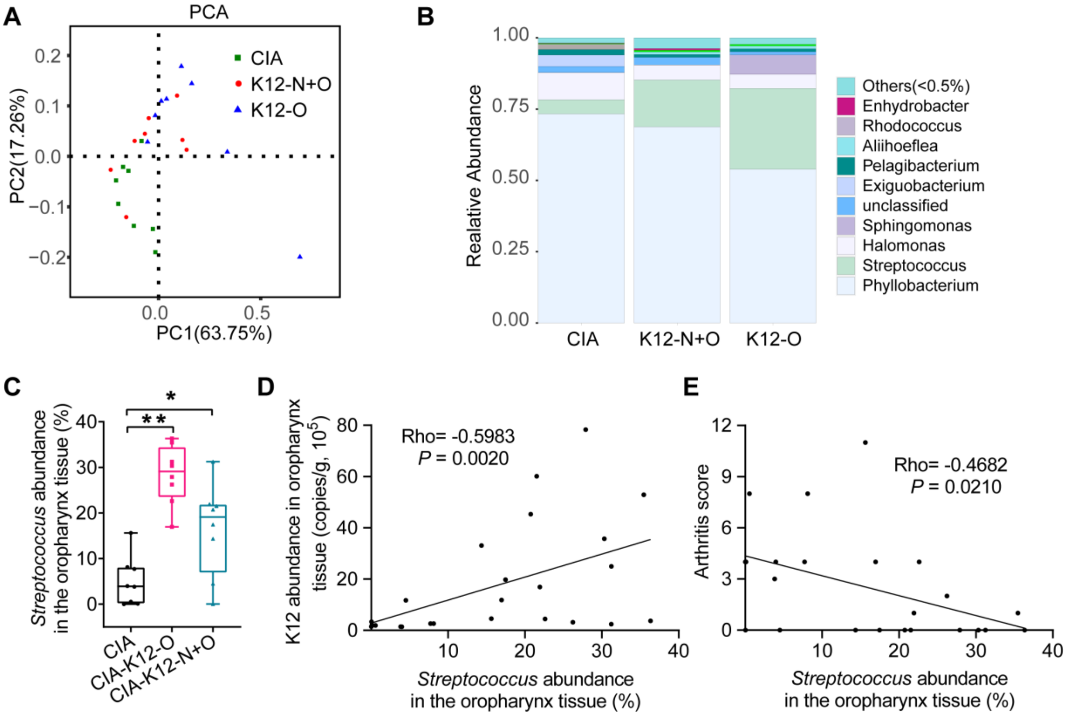

Supplementary Figure 10. 16S RNA sequencing of oropharyngeal mucosa tissues in CIA mice. (A) PCA plots of CIA mice with or without *S. salivarius* K12 treatment. N=8 in each group. **(B)** Bar plots of the top 10 most abundant genera in the oropharyngeal mucosa tissues. **(C)** The relative abundance of *Streptococcus* in the oropharyngeal mucosa tissues of CIA mice detected by 16S rRNA sequencing. n=7-8 in each group. K12-O indicates *S. salivarius* K12 administered intra-orally. K12-N+O indicates *S. salivarius* K12 administered intra-nasally and intra-orally. **(D and E)** The scatter plots depict associations of the relative abundance of *Streptococcus* with *S. salivarius* K12 amount and arthritis scores. n=24. Significance was determined using Kruskal-Wallis test followed by Dunn's multiple comparisons test (C) or Spearman's rank test (D and E). *$P < 0.05$, **$P < 0.01$.
